# Genome-wide analysis of DNA methylation in subjects with type 1 diabetes identifies epigenetic modifications associated with proliferative diabetic retinopathy

**DOI:** 10.1186/s12916-015-0421-5

**Published:** 2015-08-06

**Authors:** Elisabet Agardh, Annika Lundstig, Alexander Perfilyev, Petr Volkov, Tove Freiburghaus, Eero Lindholm, Tina Rönn, Carl-David Agardh, Charlotte Ling

**Affiliations:** Department of Clinical Sciences, Ophthalmology, Lund University, Scania University Hospital, 205 02 Malmö, Sweden; Department of Clinical Sciences, Epigenetics and Diabetes Unit, Lund University Diabetes Centre, CRC, Scania University Hospital, 205 02 Malmö, Sweden; Department of Clinical Sciences, Endocrinology, Lund University, Scania University Hospital, 205 02 Malmö, Sweden

**Keywords:** Diabetic complication, DNA methylation, Epigenetics, Inflammation, Prediction, Proliferative retinopathy, Prospective, Type 1 diabetes

## Abstract

**Background:**

Epigenetic variation has been linked to several human diseases. Proliferative diabetic retinopathy (PDR) is a major cause of vision loss in subjects with diabetes. However, studies examining the association between PDR and the genome-wide DNA methylation pattern are lacking. Our aim was to identify epigenetic modifications that associate with and predict PDR in subjects with type 1 diabetes (T1D).

**Methods:**

DNA methylation was analyzed genome-wide in 485,577 sites in blood from cases with PDR (n = 28), controls (n = 30), and in a prospective cohort (n = 7). False discovery rate analysis was used to correct the data for multiple testing. Study participants with T1D diagnosed before 30 years of age and insulin treatment within 1 year from diagnosis were selected based on 1) subjects classified as having PDR (cases) and 2) subjects with T1D who had had diabetes for at least 10 years when blood DNA was sampled and classified as having no/mild diabetic retinopathy also after an 8.7-year follow-up (controls). DNA methylation was also analyzed in a prospective cohort including seven subjects with T1D who had no/mild diabetic retinopathy when blood samples were taken, but who developed PDR within 6.3 years (converters). The retinopathy level was classified by fundus photography.

**Results:**

We identified differential DNA methylation of 349 CpG sites representing 233 unique genes including *TNF*, *CHI3L1* (also known as *YKL-40*), *CHN2*, *GIPR*, *GLRA1*, *GPX1*, *AHRR*, and *BCOR* in cases with PDR compared with controls. The majority of these sites (79 %) showed decreased DNA methylation in cases with PDR. The Natural Killer cell-mediated cytotoxicity pathway was found to be significantly (*P* = 0.006) enriched among differentially methylated genes in cases with PDR. We also identified differential DNA methylation of 28 CpG sites representing 17 genes (e.g. *AHRR*, *GIPR*, *GLRA1*, and *BCOR*) with *P* <0.05 in the prospective cohort, which is more than expected by chance (*P* = 0.0096).

**Conclusions:**

Subjects with T1D and PDR exhibit altered DNA methylation patterns in blood. Some of these epigenetic changes may predict the development of PDR, suggesting that DNA methylation may be used as a prospective marker of PDR.

**Electronic supplementary material:**

The online version of this article (doi:10.1186/s12916-015-0421-5) contains supplementary material, which is available to authorized users.

## Background

Proliferative diabetic retinopathy (PDR) and macular edema are the main causes of vision loss in subjects with diabetes [1]. Subjects with type 1 diabetes (T1D) have a higher prevalence of PDR than those with type 2 diabetes (T2D) and key risk factors for development of PDR include hyperglycemia, diabetes duration, hypertension, and genetic factors [1]. The Diabetes Control and Complications Trial has shown that intensive glycemic control reduces the incidence of PDR in subjects with T1D [2]. However, after said trial, the Epidemiology of Diabetes Interventions and Complications research group continued to follow the same subjects and found that the measure of glycemic control, HbA1c, deteriorated in the previously intensively treated group of subjects, while it improved in the conventionally treated one, so that the subsequent HbA1c levels no longer differed during the 10-year follow-up period [3]. Yet the disparity in the cumulative incidence of further progression of PDR between the groups continued to increase, indicating that mechanisms underlying the progressive alterations in retinal microvessels might be long lasting [3]. It has been hypothesized that transient peaks of hyperglycemia might be an independent risk factor for progression of retinopathy in these subjects and that hyperglycemic peaks may cause persistent epigenetic changes despite subsequent normoglycemia [4]. Epigenetic modifications, such as DNA methylation and histone modifications, influence many cellular processes including regulation of gene transcription, embryonic development, X chromosome inactivation, and genomic imprinting [5]. Altered epigenetic patterns may contribute to disease development and differential DNA methylation has been found in subjects with T1D and T2D compared with non-diabetic controls [6–10]. Epigenetic modifications may also influence the development of vascular complications in diabetic subjects and a recent study demonstrated that differential DNA methylation was associated with diabetic nephropathy [11]. However, whether epigenetic modifications are associated with PDR in subjects with T1D remains unknown. The aim of this study was therefore to analyze the genome-wide DNA methylation pattern in blood from subjects with T1D, including 28 cases with PDR and 30 controls. Using a prospective cohort, we also tested if changes in DNA methylation can be found in subjects with T1D prior to development of PDR and thereby predict the disease.

## Methods

### Study participants

Study participants with T1D, diagnosed before 30 years of age and insulin treatment within 1 year from diagnosis, were selected from the regional diabetes register (Diabetes 2000) according to the following criteria: 1) subjects with T1D for at least 10 years and classified as having no/mild diabetic retinopathy with, at most, a few microaneurysms at initiation of the study (n = 37), and 2) subjects with new-formed pathological retinal vessels classified as having PDR (n = 28). All included subjects had genomic DNA isolated from blood cells available. The degree of retinopathy was verified by available fundus photographs (EA) or by record information on vitreous hemorrhage at the time of blood sampling. The degree of retinopathy was thereafter established approximately once per year by fundus photography. After an approximately 6.3-year follow-up, seven out of 37 subjects had developed PDR, forming a prospective follow-up cohort, and were classified as converters. Additionally, after an approximately 8.7-year follow-up, 30 out of 37 subjects were still without PDR and classified as controls; the 28 subjects with PDR were classified as cases. The characteristics of cases and controls are described in Table 1 and of converters and controls in Additional file 1: Table S1. The study was approved by the Regional Ethical Committee (Lund University) and conducted in accordance with the principles of the Helsinki Declaration. All study participants gave written informed consent.

**Table 1:** Characteristics of cases with proliferative diabetic retinopathy and controls included in the genome-wide analysis of DNA methylation in blood from subjects with type 1 diabetes

Characteristics	Controls	Cases	*P* value
N (male/female)	30 (15/15)	28 (17/11)	
Age (years)*	36.1 ± 13.7	34.0 ± 8.5	1
Diabetes duration (years)	17.1 ± 9.6	21.0 ± 3.8	1 × 10^−4^
HbA1c (%)	7.1 ± 1.2	8.6 ± 1.6	2 × 10^−4^
Systolic blood pressure (mmHg)	126.7 ± 15.9	133.0 ± 21.4	0.44
Diastolic blood pressure (mmHg)	72.9 ± 9.9	77.0 ± 8.1	0.15

Data are presented a mean ± SD. *P* value based on Mann–Whitney two independent samples test. *Age at DNA sampling and fundus photography

### Genome-wide analysis of DNA methylation

The genome-wide DNA methylation analysis was performed using the Infinium HumanMethylation450 BeadChip (Illumina, San Diego, CA, USA), which contains 485,577 probes that cover 21,231 (99 %) RefSeq genes [12]. Genomic DNA (500 ng) from blood was bisulfite treated using the EZ DNA methylation kit (Zymo Research, Orange, CA, USA) and subsequently used for analysis of DNA methylation following the Infinium HD Assay Methylation Protocol (Illumina). The BeadChips’ images were captured using the Illumina iScan. All included samples showed a high quality bisulfite conversion efficiency (intensity signal >4000) and passed all GenomeStudio quality control steps based on built-in control probes for staining, hybridization, extension, and specificity. In total, 2,309 individual probes were filtered away based on mean detection *P* >0.01 and 85 Y chromosome probes were filtered away. The raw methylation data was exported from GenomeStudio and analyzed using Bioconductor and the lumi package. Here, β-values were converted to M-values (M=log^2^(β/(1-β))) [13]. Next, data was background corrected by subtracting the median M-value of built in control probes and normalized using quantile normalization [13]. The DNA methylation array data were further corrected for batch effects using COMBAT [13]. Finally, to more easily interpret the results, M-values were reconverted to β-values, which is used when describing the data and creating the figures. All identified significant CpG sites with differences in DNA methylation between PDR cases and controls were screened for cross-reactive probes in the Illumina Infinium HumanMethylation450k BeadChip [14].

### Statistical analyses

We performed a principle component analysis of the genome-wide DNA methylation data and correlated the top five principle components with PDR, gender, age, duration of diabetes, and HbA1c. To identify differences in DNA methylation between cases with PDR and controls, a logistic regression model was used, including gender, age, and duration of diabetes as covariates and DNA methylation as a quantitative variable. A false discovery rate (FDR) analysis was used to correct for multiple testing and a FDR less than 5 % (*q* <0.05) was considered significant. Significant CpG sites were analyzed in the prospective cohort using the same statistical model and covariates, and presented if *P* <0.05.

## Results

### Differential DNA methylation of specific CpG sites in cases with PDR

**Fig. 1:**
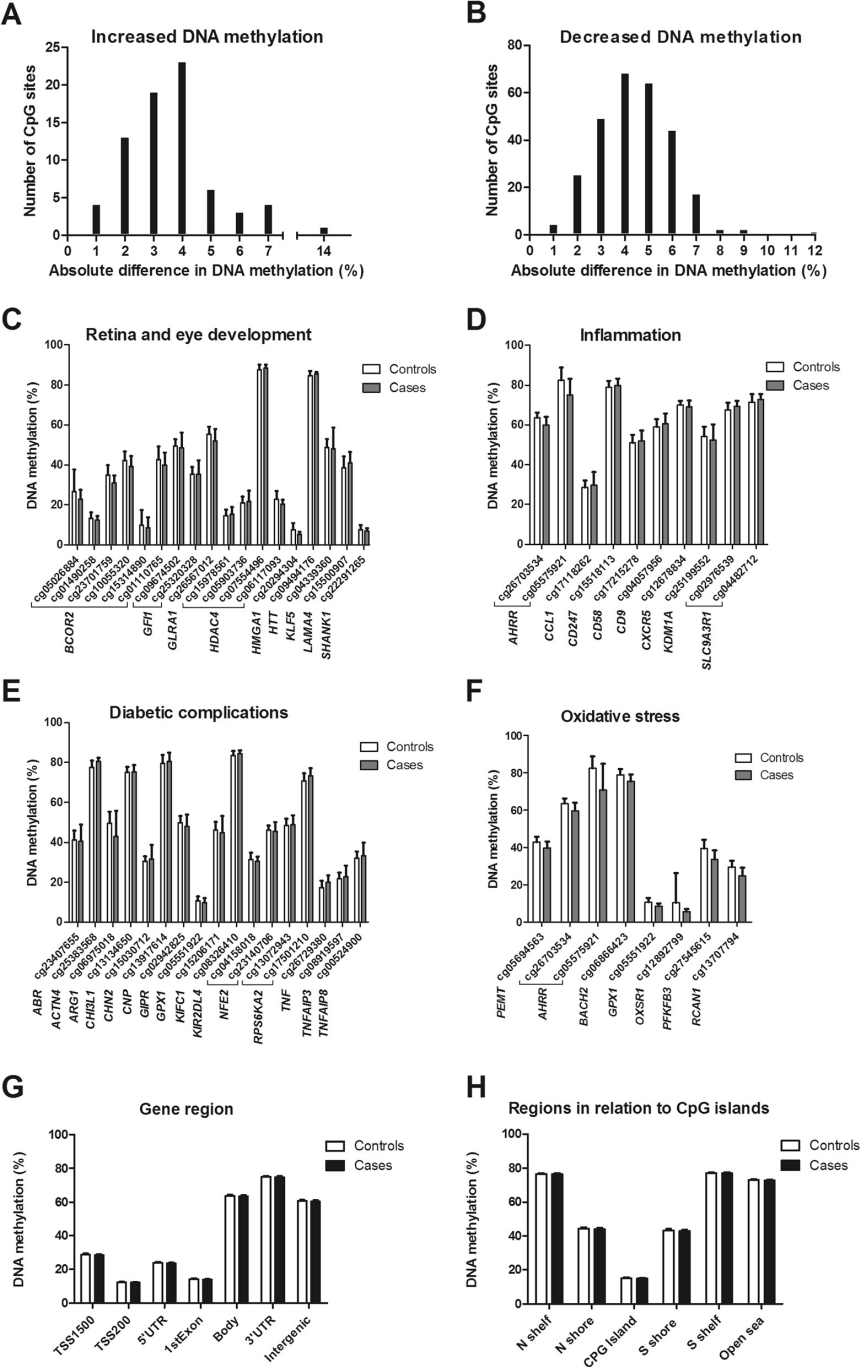
Number of CpG sites with significantly higher (**a**) or lower (**b**) DNA methylation in cases with PDR versus controls. Genes involved in retina and eye development (**c**), inflammation (**d**), diabetic complications (**e**), and oxidative stress (**f**) were among the ones with one or more CpG sites differentially methylated between cases with PDR and controls (*q* <0.05); data are presented as mean ± SD. Global DNA methylation is calculated as average DNA methylation based on all CpG sites in each region on the chip and shows no differences between cases with PDR compared with controls in mean methylation value based on gene regions (**g**) or regions in relation to nearest CpG island (**h**)

To study the epigenetic basis of PDR in subjects with T1D, DNA methylation of 480,079 CpG sites was analyzed in blood from 28 cases with PDR and 30 controls using the Infinium HumanMethylation450 BeadChip. The characteristics of the cases and controls included in the genome-wide analysis of DNA methylation are described in Table 1. Age was similar between the groups but cases with PDR had longer diabetes duration and higher HbA1c levels than controls (Table 1). To examine the impact of these factors on DNA methylation, we performed a principle component analysis of the genome-wide DNA methylation data and correlated the top five principle components with PDR, gender, age, duration of diabetes, and HbA1c levels. While PDR, gender, age, and duration of diabetes showed significant correlations with one or several of the top five principle components (*P* <0.05; Additional file 2: Table S2); HbA1c was not significant in this analysis. Based on this result, we included gender, age, and duration of diabetes as covariates in the regression model where the association between PDR and DNA methylation was studied. After correcting for multiple testing using a FDR analysis, we identified 349 CpG sites representing 233 unique genes that were differentially methylated between cases with PDR and controls (*q* <0.05; Additional file 3: Table S3). We found decreased DNA methylation in 276 sites (79 %) and increased DNA methylation in 73 sites (21 %) in PDR cases. The absolute differences in DNA methylation ranged from 0.08 % to 13.5 %, representing a fold change of 0.549 to 1.231 and a 45.1 % decrease to a 23.1 % increase in methylation in PDR cases compared with controls (Fig. 1a,b and Additional file 3: Table S3). Based on a literature search, genes with known functions in retina and eye development (*BCOR*, *GFI1*, *GLRA1*, *HDAC4*, *HMGA1*, *HTT, KLF5, LAMA4*, and *SHANK1*) [15], inflammation (*AHRR*, *CCL1*, *CD247*, *CD58*, *CD9*, *CXCR5*, *KDM1A*, and *SLC9A3R1*) [16], diabetic complications (*ACTN4*, *ARG1*, *CHI3L1*, *CHN2*, *CNP*, *GIPR*, *GPX1*, *KIFC1*, *NFE2*, *PEMT*, *RPS6KA2*, *TNF*, *TNFAIP3*, and *TNFAIP8*) [17], and oxidative stress (*AHRR*, *BACH2*, *GPX1*, *OXSR1*, *PFKFB3*, and *RCAN1*) [18] were among the genes that showed differential DNA methylation in blood from T1D subjects with PDR (Fig. 1c–f). Some of these genes are represented in several of the analyzed groups.

We then performed a pathway analysis of the 233 genes with one or more CpG sites differentially methylated between cases with PDR and controls using WebGestalt [19]. We found the KEGG pathway “Natural Killer cell mediated cytotoxicity” to be significantly enriched in the PDR cases (adjusted *P* = 0.006), including eight genes: *KLRD1*, *TNF*, *CD247*, *PIK3CD*, *GZMB*, *NFATC1*, *PRF1*, and *KIR2DL4* (Table 2).

**Table 2:** Pathway analysis of genes with CpG sites differentially methylated in cases with PDR versus controls

KEGG pathway: Natural Killer cell mediated cytotoxicity (*P* = 0.0001; *P*_adj_ = 0.0063)		
Total # of genes in pathway: 121	Expected # of genes: 1.48	Observed # of genes: 8
Gene symbol	Description	
*KLRD1*	Killer cell lectin-like receptor subfamily D, member 1	
*TNF*	Tumor necrosis factor	
*CD247*	CD247 molecule	
*PIK3CD*	Phosphatidylinositol-4,5-bisphosphate 3-kinase, catalytic subunit delta	
*GZMB*	Granzyme B (granzyme 2, cytotoxic T-lymphocyte-associated serine esterase 1)	
*NFATC1*	Nuclear factor of activated T-cells, cytoplasmic, calcineurin-dependent 1	
*PRF1*	Perforin 1 (pore forming protein)	
*KIR2DL4*	Killer cell immunoglobulin-like receptor, two domains, long cytoplasmic tail, 4	

### Global DNA methylation in blood from cases with PDR and controls

To evaluate the global methylome in blood from cases with PDR and controls, we calculated the average level of DNA methylation in the two groups based on either the functional genome distribution (Fig. 1g) or the CpG content and neighborhood context (Fig. 1h). The results showed no significant differences in global DNA methylation between PDR cases and controls after FDR correction (Fig. 1g,h, Additional file 4: Table S4). Nevertheless, the average level of DNA methylation was high within the gene body, 3′UTR, and in intergenic regions (60–75 %), whereas it was lower in TSS1500 and 5′UTR (23–29 %) and lowest within TSS200 and the first exon (12–14 %; Fig. 1g). In relation to CpG islands, the average methylation was low within the CpG island (15 %), intermediate in the shore regions (43–44 %), and high in the shelf regions and open sea (73–77 %; Fig. 1h).

### DNA methylation as a prospective marker of PDR

We next tested if differences in DNA methylation may be used as prospective markers of PDR in subjects with T1D. Here, we used DNA from blood from T1D subjects with no/mild diabetic retinopathy when the blood samples were taken, but who developed PDR within approximately 6 years (converters), and compared the degree of DNA methylation in these converters with methylation in control subjects who did not develop PDR (controls; Additional file 1: Table S1). In this prospective cohort there was no significant differences in diabetes duration between converters and controls, but the converters had significantly lower age and higher HbA1c levels (*P* <0.05; Additional file 1: Table S1) compared to the controls. We examined if any of the 349 CpG sites showing differential DNA methylation in the PDR cases versus controls (Additional file 3: Table S3) also show differential DNA methylation in the prospective cohort. We found 28 sites representing 17 unique genes including *BCOR*, *GIPR*, and *GLRA1* that showed differential DNA methylation in the same direction in the prospective cohort as well as in the case–control cohort with *P* ≤0.05 (Table 3). This is more than expected by chance based on a χ^2^ test (*P* = 0.0096). However, the FDR analysis shows that these sites are only nominally significant in the prospective cohort (Table 3). The majority of these CpG sites (93 %) exhibit lower methylation in the converters compared with the controls.

**Table 3:** CpG sites showing significant differences in DNA methylation between cases with PDR and controls (FDR *q* <5 %, Additional file 3: Table S3), that are also nominally differentially methylated in the same direction in the converters of the prospective cohort at *P* <0.05

		Location in relation to:			DNA Methylation (%)				
Probe ID	Chr	Nearest gene	Gene region	CpG Island	Controls	Converters	Difference	*P* value	*q* value
cg26703534	5	*AHRR*	Body	S Shelf	63.6 ± 2.6	59.9 ± 4.1	−3.7	9.5 × 10^−4^	0.17
cg05575921	5	*AHRR*	Body	N Shore	82.4 ± 6.3	75.0 ± 8.1	−7.4	2.1 × 10^−3^	0.18
cg07339236	20	*ATP9A*	Body	Open sea	18.0 ± 2.5	15.6 ± 1.6	−2.4	0.013	0.35
cg05026884	X	*BCOR*	5′UTR	Island	26.5 ± 11.1	22.7 ± 4.7	−3.8	4.2 × 10^−3^	0.21
cg01490258	X	*BCOR*	5′UTR	N Shore	13.2 ± 2.9	12.4 ± 2.0	−0.8	0.010	0.33
cg23701759	X	*BCOR*	5′UTR	Island	34.9 ± 4.9	30.9 ± 3.7	−4.0	0.014	0.35
cg10055320	X	*BCOR*	5′UTR	N Shore	42.2 ± 4.4	39.2 ± 5.1	−3.0	0.029	0.50
cg00507154	19	*C19orf76*	1^st^ Exon; 5′UTR	Island	70.2 ± 4.6	67.7 ± 2.6	−2.5	0.030	0.50
cg08601628	8	*C8orf74*	Body	Open sea	82.5 ± 3.0	80.6 ± 3.2	−1.9	0.022	0.43
cg12993916	6	*DTNBP1*	Body	Open sea	78.9 ± 3.0	76.8 ± 3.7	−2.1	0.015	0.35
cg03636183	19	*F2RL3*	Body	N Shore	68.9 ± 4.4	65.1 ± 4.7	−3.8	9.3 × 10^−3^	0.32
cg02942825	19	*GIPR*	3′UTR	S Shore	49.8 ± 3.2	48.0 ± 5.8	−1.8	0.050	0.62
cg17808910	7	*GLCCI1*	Body	S Shelf	73.7 ± 3.9	75.5 ± 4.6	1.8	0.048	0.62
cg26567012	5	*GLRA1*	3′UTR	Open sea	55.4 ± 3.8	52.0 ± 6.0	−3.4	0.030	0.50
cg01010839	10	*NET1*	TSS1500; Body	N Shore	71.2 ± 4.3	70.2 ± 2.2	−1.0	0.015	0.35
cg24796663	6	*PHF1*	TSS1500	N Shore	12.9 ± 1.8	11.3 ± 1.9	−1.6	1.5 × 10^−3^	0.17
cg11224624	8	*PLEC1*	Body	Island	2.1 ± 0.3	2.3 ± 0.3	0.2	0.033	0.52
cg22291265	19	*SHANK1*	Body	Open sea	7.5 ± 2.3	6.9 ± 1.3	−0.6	0.050	0.62
cg06589051	2	*TGFBRAP1*	TSS1500	S Shore	72.4 ± 2.0	70.2 ± 4.1	−2.2	0.040	0.61
cg08597832	8	*TOP1MT*	Body	N Shore	82.1 ± 2.5	79.8 ± 2.7	−2.3	0.020	0.41
cg11166303	2	*TSSC1*	Body	N Shore	67.8 ± 7.5	61.2 ± 8.5	−6.6	4.0 × 10^−3^	0.21
cg09577317	8		Intergenic	N Shore	83.9 ± 1.6	81.0 ± 1.2	−2.9	1.7 × 10^−4^	0.058
cg07948143	14		Intergenic	Open sea	75.5 ± 3.2	71.0 ± 4.6	−4.5	2.9 × 10^−3^	0.20
cg24058013	18		Intergenic	Island	89.8 ± 1.0	87.7 ± 1.8	−2.1	5.4 × 10^−3^	0.24
cg03889263	3		Intergenic	Open sea	20.0 ± 3.9	15.9 ± 2.6	−4.1	6.9 × 10^−3^	0.27
cg05546763	14		Intergenic	Island	82.0 ± 2.0	80.6 ± 2.3	−1.4	0.016	0.36
cg15342087	6		Intergenic	Open sea	81.1 ± 2.1	80.2 ± 2.2	−0.9	0.046	0.62
cg06126421	6		Intergenic	Open sea	69.2 ± 6.2	69.0 ± 4.4	−0.2	0.050	0.62

Data are presented a mean ± SD

### Cross-reactive probes

As a quality control, we further screened the 349 sites with significant differences in DNA methylation between PDR cases and controls for cross-reactive probes in the Illumina Infinium HumanMethylation450k BeadChip [14]. Only one of the 349 probes had a perfect match elsewhere in the genome and 13 probes matched for 47–49 out of 50 bases (Additional file 5: Table S5).

### Association between HbA1c levels and DNA methylation in blood

We finally tested if HbA1c was associated with differential DNA methylation of individual CpG sites in the control cohort including subjects with T1D without PDR. Gender, age, and diabetes duration were included as covariates in the linear regression model. However, after correction for multiple testing using FDR, no significant association between HbA1c and DNA methylation was found (*q* = 0.99). Additionally, HbA1c was only associated with methylation of 14,522 CpG sites at *P* <0.05, which is much less than expected by chance.

Even though HbA1c was not significantly associated with DNA methylation based on this linear regression analysis, nor based on the principle component analysis, we tested to include HbA1c in the regression model for the association between DNA methylation and PDR, as there was a significant difference in HbA1c between PDR cases and controls. Although not genome-wide significant (*q* = 0.08–0.38), the 349 CpG sites detected to be differentially methylated between PDR cases and controls in our main analysis (*q* <0.05; Additional file 3: Table S3) all still showed a strong nominal association (*P* = 4.0 × 10^−7^ to 7.6 × 10^−3^) after inclusion of HbA1c in the model (Additional file 6: Table S6). Thereby, our results support that adjustment for HbA1c has no major effect on the association between PDR and DNA methylation, but it makes the study less well powered since the size of our study only supports the inclusion of up to four variables in the regression analysis in order to maintain statistical power. Additionally, when including HbA1c in the model we found increased standard errors in the regression analysis of all 349 CpG sites (Additional file 6: Table S6), indeed suggesting that this additional variable makes the model less stable.

## Discussion

In the present study, we demonstrate for the first time that differential DNA methylation in/near 233 unique genes is associated with PDR in a case–control cohort. In a prospective cohort, we also found epigenetic markers that can predict PDR. Our study suggests that DNA methylation in blood can be used as a predictive biomarker of PDR in subjects with T1D. However, replication studies are needed to verify our results.

Diabetic retinopathy is a common microvascular complication and, in its severe forms, the leading cause of blindness among working-aged adults in the western world [1]. In PDR, ischemia is preceding formation of new vessels, which untreated can result in vitreous hemorrhage and retinal detachment. Inflammation does also play a key role in development of microvascular diabetic complications, including PDR [17]. Subjects with T1D have a relatively high prevalence of diabetic retinopathy compared with subjects with T2D and it is well established that HbA1c levels, diabetes duration, and hypertension increase the risk for PDR [20]. Previous studies have tried to identify biomarkers that can be used to clinically predict diabetic retinopathy in subjects with diabetes [21]. However, so far, limited breakthroughs have taken place [22–24]. In our study, we chose to identify markers, not for diabetic retinopathy per se but for the most severe consequence of ischemic retinopathy, i.e. PDR. Since diabetes duration and degree of non-proliferative retinopathy are risk factors for PDR development, we aimed at including control subjects with as low risk as possible. It was not possible to identify a sufficient number of control subjects with at least 10 years of diabetes who were also completely without microaneurysms, but as occasional microaneurysms may come and go we included subjects with a few microaneurysms. Using a longitudinal approach, we could conclude that 81 % (30 out of 37 subjects) of these subjects still were without PDR approximately 8 years later and they were assigned as controls. Progression to PDR occurred in seven subjects (19 %) and were assigned converters. Those subjects differed from both cases and controls by younger age and poorer metabolic control measured by HbA1c. Those characteristics are associated with a high risk for the rapid development of severe PDR, also known as florid retinopathy [25], which results in severe visual impairment or blindness if not properly treated in time. Based on our study design, we have been able not only to identify epigenetic markers associated with PDR but also predictive of the most severe form of diabetic retinopathy in T1D.

We identified 349 CpG sites representing 233 genes with altered DNA methylation levels in T1D subjects with PDR. The majority of these sites (79 %) showed decreased DNA methylation in cases with PDR. A lower methylation level may be associated with higher expression as DNA methylation has been shown to repress the binding of transcription factors to promoter regions and attract proteins such as transcriptional co-repressors and histone deacetyltransferases associated with a dense chromatin structure and inactive genes [5]. Although it would have been interesting to also study gene expression in the present case–control cohort, this was not possible since only DNA was extracted when blood was collected. Nevertheless, DNA methylation in blood can quite easily be used as a biomarker that predicts disease and the epigenetic alterations found in the present study suggest that this may be possible in subjects with T1D.

Several identified genes with altered DNA methylation in cases with PDR, such as *TNF*, *CHI3L1*, and *CHN2*, encode proteins with previous known function in diabetic complications and/or diabetic retinopathy [17]. *TNF*, which encodes tumor necrosis factor, exhibits decreased DNA methylation in cases with PDR. Tumor necrosis factor is a proinflammatory cytokine that is elevated in serum from subjects with PDR [26]. Epigenetic modifications of *TNF* in T1D subjects may subsequently regulate the expression of *TNF* and thereby contribute to inflammation in diabetic retinopathy. *CHI3L1*, also known as *YKL-40*, encodes an inflammatory glycoprotein involved in endothelial dysfunction and cardiovascular disease [27]. We found decreased DNA methylation of *CHI3L1* in cases with PDR, which may contribute to increased expression of this gene. Interestingly, serum levels of YKL-40 were elevated in subjects with diabetic retinopathy [28] and ischemia was associated with higher *YKL-40* mRNA levels in carotid plaque [29]. As ischemia precedes PDR, it may also affect methylation and/or expression of *CHI3L1* in subjects with diabetic retinopathy. Cases with PDR also exhibited decreased DNA methylation of *CHN2*, which encodes chimerin 2, a protein known to affect proliferation and migration of smooth muscle cells. Interestingly, a SNP in an intron of *CHN2* was recently associated with diabetic retinopathy in Chinese people with T2D, further supporting a role for this gene in the development of diabetic retinopathy [30].

The largest absolute difference in DNA methylation between PDR cases and controls was 13.5 %, which represents a fold change of 1.22. Our epigenetic data are in line with previous genetic studies where the odds ratio of most genetic variants associated with PDR has been modest [31]. However, we identified more than 200 genes with differential DNA methylation in PDR cases after correction for multiple testing and it is possible that modest changes of multiple genes together contribute to the disease phenotype. The present study is, to our knowledge, the first one to analyze DNA methylation genome-wide in subjects with PDR. However, a previous study by Bell et al. [11] also reported absolute differences in DNA methylation of approximately 10 % when DNA methylation of approximately 27,000 sites was analyzed in blood from subjects with diabetic nephropathy.

In order to assess if epigenetic modifications may be used as biomarkers that predict PDR, we analyzed DNA methylation in a prospective cohort. Differential DNA methylation may predict PDR as several epigenetic modifications identified in cases with PDR were also found in our prospective cohort. *BCOR* encoding BCL6 co-repressor has the most CpG sites differentially methylated in both the case–control cohort and in the prospective cohort. While these data suggest that epigenetic modifications of *BCOR* may be used to predict PDR, larger prospective studies are needed to further validate our findings. *GIPR*, encoding glucose-dependent insulinotropic polypeptide receptor, also showed decreased DNA methylation in our prospective cohort as well as in the cases with PDR, and may subsequently be used as a candidate gene that predicts PDR. Interestingly, a previous study found increased expression of the *GIPR* in the retinas of streptozotocin-induced diabetic rats [32]. However, whether expression of the *GIPR* is increased in the human retina of diabetic subjects remains unknown and will be ethically difficult to analyze. The prospective cohort also confirmed demethylation of the aryl hydrocarbon receptor repressor (*AHRR*) in PDR cases, a locus which has been repeatedly shown to associate with smoking [33–35]. Unfortunately, we do not have access to smoking status in our study from when samples were collected and hence we cannot rule out that altered smoking habits between the groups may affect this association.

Our study supports a role of epigenetic variation in the pathogenesis of PDR. Furthermore, the heritability of PDR has been estimated to 27 % and genetic variants may thereby affect the risk for PDR [36]. However, although genome-wide association studies have been performed to identify genetic variants associated with PDR, the outcome from these studies was limited [36–38]. It is possible that genetic variants interact with epigenetic variation to increase the risk for disease [39, 40]. However, further genome-wide studies are needed to dissect whether interactions between genetics and epigenetics play a role in PDR.

## Conclusion

Together, our study is the first to identify epigenetic modifications in T1D subjects with PDR. Our data suggest that differential DNA methylation may contribute to the pathogenesis of PDR in subjects with T1D and that DNA methylation in blood may be used as a predictive biomarker for this severe type of diabetic retinopathy. However, replication is needed to establish whether the findings really represent a novel biomarker of retinopathy risk.

## Additional files

Additional file 1: Table S1. Characteristics of type 1 diabetic subjects converting to proliferative diabetic retinopathy (converters) and controls. (DOC 35 kb) Additional file 2: Table S2.
*P* values for correlations of the top five principle components for the DNA methylation data with proliferative diabetic retinopathy, gender, age, duration of diabetes, and HbA1 levels. (DOC 30 kb) Additional file 3: Table S3. CpG sites significantly different between type 1 diabetic subjects with (cases) and without (controls) proliferative diabetic retinopathy (false discovery rate <5 %). (DOC 554 kb) Additional file 4: Table S4. Average level of DNA methylation for regions in relation to nearest gene or CpG islands, in type 1 diabetic subjects with (cases) or without (controls) proliferative diabetic retinopathy. (DOC 42 kb) Additional file 5: Table S5. The 14 cross-reactive probes with number of bases matched to cross-reactive target(s). (DOC 43 kb) Additional file 6: Table S6. Associations between proliferative diabetic retinopathy (PDR) and DNA methylation when adding HbA1c to the logistic regression model for all the 349 CpG sites significantly different between type 1 diabetic subjects with (cases) and without (controls) PDR based on a false discovery rate of <5 % in the main analysis. Beta coefficients and standard error of the mean (SE) are presented for the regression analysis both with and without HbA1c in the model. (DOC 631 kb)

## Abbreviations

FDR: False discovery rate
PDR: Proliferative diabetic retinopathy
T1D: Type 1 diabetes
T2D: Type 2 diabetes
