# Supplementary material for: Genome-wide analysis of DNA methylation in subjects with type 1 diabetes identifies epigenetic modifications associated with proliferative diabetic retinopathy
Source: BMC Med. 2015 Aug 6;13:182. doi: 10.1186/s12916-015-0421-5 (PMC4527111; doi:10.1186/s12916-015-0421-5)
Supplement: Additional file 1: Table S1. — Characteristics of type 1 diabetic subjects converting to proliferative diabetic retinopathy (converters) and controls. (DOC 35 kb) [file 12916_2015_421_MOESM1_ESM.doc]

Additional file 1: **Table S1**. Characteristics of type 1 diabetic subjects converting to PDR (converters) and controls.

| **Characteristics** | **Controls** | **Converters** | ***P*-value** |
| --- | --- | --- | --- |
| *N* (male/female) | 30 (15/15) | 7 (3/4) |  |
| Age (years)* | 36.1 ± 13.7 | 21.7 ± 2.0 | 0.0007 |
| Diabetes duration at baseline (years) | 17.1 ± 9.6 | 13.1 ± 2.5 | 0.4 |
| HbA1c at baseline (%) | 7.1 ± 1.2 | 10.4 ± 1.2 | 0.0001 |
| Age at follow-up (years)^#^ | 44.8 ± 14.8 | 28.0 ± 2.1 | 0.002 |
| Systolic BP (mm Hg) | 126.7 ± 15.9 | 125.0 ± 18.7 | 0.5 |
| Diastolic BP (mm Hg) | 72.9 ± 9.9 | 73.6 ± 11.1 | 0.4 |

Data are presented a mean ± SD. *P*-value of Mann-Whitney two independent samples test.

*Age at DNA sampling and first fundus photography.

^#^ Age at last fundus photography.

BP: blood pressure.
