# Supplementary material for: Genome-wide analysis of DNA methylation in subjects with type 1 diabetes identifies epigenetic modifications associated with proliferative diabetic retinopathy
Source: BMC Med. 2015 Aug 6;13:182. doi: 10.1186/s12916-015-0421-5 (PMC4527111; doi:10.1186/s12916-015-0421-5)
Supplement: Additional file 2: Table S2. — P values for correlations of the top five principle components for the DNA methylation data with proliferative diabetic retinopathy, gender, age, duration of diabetes, and HbA1 levels. (DOC 30 kb) [file 12916_2015_421_MOESM2_ESM.doc]

| Principle component | PDR | Gender | Age | Duration of diabetes | HbA1c |
| --- | --- | --- | --- | --- | --- |
| 1 | 0.19 | 0.026 | <0.0001 | 0.06 | 0.12 |
| 2 | 0.40 | <0.0001 | 0.89 | 0.59 | 0.96 |
| 3 | 0.11 | 0.91 | <0.0001 | 0.009 | 0.43 |
| 4 | 0.42 | 0.89 | 0.06 | 0.04 | 0.30 |
| 5 | 0.04 | 0.91 | <0.0001 | 0.0007 | 0.16 |

Additional file 2: **Table S2.** *P*-values for correlations of the top five principle components for the DNA methylation data with PDR, gender, age, duration of diabetes and HbA1 levels.
