# Supplementary material for: Genome-wide analysis of DNA methylation in subjects with type 1 diabetes identifies epigenetic modifications associated with proliferative diabetic retinopathy
Source: BMC Med. 2015 Aug 6;13:182. doi: 10.1186/s12916-015-0421-5 (PMC4527111; doi:10.1186/s12916-015-0421-5)
Supplement: Additional file 4: Table S4. — Average level of DNA methylation for regions in relation to nearest gene or CpG islands, in type 1 diabetic subjects with (cases) or without (controls) proliferative diabetic retinopathy. (DOC 42 kb) [file 12916_2015_421_MOESM4_ESM.doc]

Additional file 4: **Table S4**. Average level of DNA methylation for regions in relation to nearest gene or CpG islands, in type 1 diabetic subjects with (cases) or without (controls) proliferative diabetic retinopathy.

| **Region in relation to**  **Nearest Gene** | **DNA Methylation (%)** | | |
| --- | --- | --- | --- |
|  | **Controls** | **Cases** | ***p*-value** |
| TSS1500 | 28.8 ± 0.7 | 28.4 ± 0.7 | 0.06 |
| TSS200 | 12.3 ± 0.4 | 12.1 ± 0.3 | 0.13 |
| 5'UTR | 23.9 ± 0.7 | 23.6 ± 0.6 | 0.09 |
| 1stExon | 14.2 ± 0.5 | 14.0 ± 0.4 | 0.16 |
| Body | 63.8 ± 0.7 | 63.5 ± 0.8 | 0.10 |
| 3'UTR | 75.2 ± 0.6 | 74.9 ± 0.7 | 0.17 |
| Intergenic | 60.8 ± 0.7 | 60.5 ± 0.8 | 0.13 |
| **Region in relation to**  **CpG Islands** | **DNA Methylation (%)** | | |
|  | **Controls** | **Cases** | ***p*-value** |
| N Shelf | 76.7 ± 0.6 | 76.5 ± 0.6 | 0.21 |
| N Shore | 44.4 ± 0.8 | 44.0 ± 0.9 | 0.05 |
| Island | 15.0 ± 0.5 | 14.9 ± 0.4 | 0.14 |
| S Shore | 43.4 ± 0.8 | 42.9 ± 0.9 | 0.04 |
| S Shelf | 77.2 ± 0.6 | 77.1 ± 0.6 | 0.25 |
| Open Sea | 73.0 ± 0.6 | 72.8 ± 0.7 | 0.20 |

Data are presented as mean ± SD.
