# Supplementary material for: Genome-wide analysis of DNA methylation in subjects with type 1 diabetes identifies epigenetic modifications associated with proliferative diabetic retinopathy
Source: BMC Med. 2015 Aug 6;13:182. doi: 10.1186/s12916-015-0421-5 (PMC4527111; doi:10.1186/s12916-015-0421-5)
Supplement: Additional file 5: Table S5. — The 14 cross-reactive probes with number of bases matched to cross-reactive target(s). (DOC 43 kb) [file 12916_2015_421_MOESM5_ESM.doc]

Additional file 5: **Table S5.** The 14 cross-reactive probes with number of bases matched to

cross-reactive target(s).

| **TargetID** | **47** | **48** | **49** | **50** |
| --- | --- | --- | --- | --- |
| cg00054352 | 0 | 0 | 1 | 0 |
| cg00805874 | 0 | 1 | 0 | 0 |
| cg02407068 | 0 | 5 | 0 | 0 |
| cg07948143 | 2 | 2 | 1 | 0 |
| cg08326410 | 0 | 0 | 0 | 1 |
| cg08975528 | 0 | 0 | 1 | 0 |
| cg09788778 | 0 | 0 | 1 | 0 |
| cg12688265 | 0 | 1 | 0 | 0 |
| cg13645242 | 0 | 0 | 1 | 0 |
| cg17278295 | 1 | 0 | 0 | 0 |
| cg24058013 | 0 | 3 | 3 | 0 |
| cg25470384 | 0 | 0 | 1 | 0 |
| cg26101890 | 4 | 1 | 0 | 0 |
| cg26181840 | 2 | 0 | 0 | 0 |
