# Supplementary material for: Genome-wide analysis of DNA methylation in subjects with type 1 diabetes identifies epigenetic modifications associated with proliferative diabetic retinopathy
Source: BMC Med. 2015 Aug 6;13:182. doi: 10.1186/s12916-015-0421-5 (PMC4527111; doi:10.1186/s12916-015-0421-5)
Supplement: Additional file 6: Table S6. — Associations between proliferative diabetic retinopathy (PDR) and DNA methylation when adding HbA1c to the logistic regression model for all the 349 CpG sites significantly different between type 1 diabetic subjects with (cases) and without (controls) PDR based on a false discovery rate of <5 % in the main analysis. Beta coefficients and standard error of the mean (SE) are presented for the regression analysis both with and without HbA1c in the model. (DOC 631 kb) [file 12916_2015_421_MOESM6_ESM.doc]

Additional file 6: **Table S6**. Associations between proliferative diabetic retinopathy (PDR) and DNA methylation when adding HbA1c to the logistic regression model for all the 349 CpG sites significantly different between type 1 diabetic subjects with (cases) and without (controls) PDR based on FDR < 5% in the main analysis. Beta coefficients and standard error of the mean (SE) are presented for the regression analysis both with and without HbA1c in the model.

|  |  | **Location in relation to:** | | | |  | **DNA Methylation (%)** | | | | | **Without HbA1c** | **Including HbA1c** |
| --- | --- | --- | --- | --- | --- | --- | --- | --- | --- | --- | --- | --- | --- |
| **Probe ID** | **Chr** | | **Nearest Gene** | **Gene region** | **CpG Island** | **Controls** | | **Cases** | **Difference** | ***p*-value** | ***q-*value** | **Beta ± SE** | **Beta ± SE** |
| cg11133963 | 17 | | *ABI3* | Body | S Shore | 76.1 ± 3.2 | | 79.6 ± 2.0 | 3,5 | 1.41x10^-04^ | 0.179 | 0.3±0.062 | 0.288±0.071 |
| cg23407655 | 17 | | *ABR* | Body | Open sea | 41.3 ± 4.5 | | 35.8 ± 5.4 | -5,5 | 5.93x10^-04^ | 0.208 | -0.384±0.087 | -0.356±0.101 |
| cg25383568 | 19 | | *ACTN4* | Body | CpG Island | 77.5 ± 3.5 | | 81.6 ± 3.0 | 4,1 | 2.27x10^-04^ | 0.185 | 0.381±0.082 | 0.361±0.094 |
| cg07093324 | 2 | | *ACTR3* | Body | S Shelf | 23.6 ± 2.6 | | 20.1 ± 3.4 | -3,5 | 2.61x10^-05^ | 0.135 | -0.382±0.072 | -0.372±0.083 |
| cg27287951 | 7 | | *ADAP1* | Body | S Shore | 87.2 ± 3.8 | | 91.4 ± 2.6 | 4,2 | 3.72x10^-05^ | 0.141 | 0.662±0.137 | 0.679±0.158 |
| cg09043403 | 6 | | *AGPAT4* | Body | Open sea | 82.0 ± 1.8 | | 80.2 ± 1.4 | -1,8 | 2.08x10^-04^ | 0.182 | -0.19±0.041 | -0.193±0.047 |
| cg09513758 | 1 | | *AHCYL1* | TSS1500 | N Shore | 79.0 ± 1.4 | | 76.9 ± 2.0 | -2,1 | 1.74x10^-04^ | 0.181 | -0.198±0.04 | -0.195±0.047 |
| cg05575921 | 5 | | *AHRR* | Body | N Shore | 82.4 ± 6.3 | | 70.8 ± 13.9 | -11,6 | 6.99x10^-03^ | 0.332 | -0.998±0.222 | -0.625±0.234 |
| cg26703534 | 5 | | *AHRR* | Body | S Shelf | 63.6 ± 2.6 | | 59.6 ± 4.3 | -4 | 3.81x10^-04^ | 0.197 | -0.293±0.055 | -0.233±0.062 |
| cg17278295 | 19 | | *AP3D1* | Body | CpG Island | 82.3 ± 3.1 | | 85.4 ± 2.1 | 3,1 | 9.41x10^-05^ | 0.169 | 0.381±0.078 | 0.369±0.09 |
| cg06975018 | 6 | | *ARG1* | TSS200 | Open sea | 49.5 ± 5.8 | | 40.8 ± 6.9 | -8,7 | 5.65x10^-05^ | 0.147 | -0.492±0.108 | -0.523±0.125 |
| cg15258980 | 2 | | *ARHGAP25* | TSS1500 | Open sea | 15.9 ± 1.9 | | 13.5 ± 1.8 | -2,4 | 1.67x10^-04^ | 0.180 | -0.284±0.064 | -0.294±0.074 |
| cg25887844 | 2 | | *ARHGAP25* | TSS1500 | Open sea | 19.8 ± 2.4 | | 17.3 ± 2.2 | -2,5 | 1.61x10^-04^ | 0.180 | -0.293±0.061 | -0.281±0.07 |
| cg15188939 | 15 | | *ARIH1* | Body | Open sea | 74.5 ± 4.0 | | 77.4 ± 3.3 | 2,9 | 6.17x10^-04^ | 0.210 | 0.286±0.06 | 0.247±0.069 |
| cg05204104 | 2 | | *ARL4C* | 3'UTR;1stExon | N Shore | 39.1 ± 6.4 | | 44.9 ± 5.7 | 5,8 | 2.71x10^-05^ | 0.135 | 0.447±0.089 | 0.456±0.103 |
| cg15016771 | 2 | | *ARL4C* | 3'UTR;1stExon | N Shore | 21.6 ± 4.5 | | 25.3 ± 4.2 | 3,6 | 1.72x10^-04^ | 0.181 | 0.382±0.087 | 0.394±0.101 |
| cg01901332 | 11 | | *ARRB1* | Body | Open sea | 67.4 ± 4.4 | | 63.1 ± 4.4 | -4,3 | 1.33x10^-04^ | 0.179 | -0.363±0.067 | -0.308±0.076 |
| cg12041266 | 11 | | *ARRB1* | Body | Open sea | 76.8 ± 2.2 | | 74.1 ± 2.5 | -2,7 | 6.90x10^-05^ | 0.160 | -0.24±0.05 | -0.248±0.057 |
| cg26337070 | 2 | | *ATOH8* | Body | Open sea | 68.0 ± 6.7 | | 62.9 ± 7.8 | -5,1 | 1.18x10^-03^ | 0.229 | -0.457±0.102 | -0.382±0.116 |
| cg07339236 | 20 | | *ATP9A* | Body | Open sea | 18.0 ± 2.5 | | 14.7 ± 2.1 | -3,3 | 2.24x10^-05^ | 0.131 | -0.387±0.069 | -0.36±0.079 |
| cg19790640 | 8 | | *BAALC* | Body;TSS1500 | S Shore | 18.5 ± 2.7 | | 15.6 ± 1.9 | -2,9 | 1.06x10^-04^ | 0.175 | -0.319±0.07 | -0.332±0.081 |
| cg06866423 | 6 | | *BACH2* | 5'UTR | Open sea | 79.0 ± 3.0 | | 75.4 ± 3.7 | -3,6 | 2.94x10^-04^ | 0.191 | -0.364±0.076 | -0.331±0.088 |
| cg06317348 | 14 | | *BCL11B* | Body | CpG Island | 18.7 ± 2.9 | | 16.1 ± 2.3 | -2,6 | 3.18x10^-04^ | 0.193 | -0.317±0.07 | -0.303±0.081 |
| cg01110765 | X | | *BCOR* | 5'UTR | N Shore | 42.6 ± 6.6 | | 37.2 ± 7.4 | -5,4 | 3.11x10^-05^ | 0.137 | -0.457±0.096 | -0.487±0.111 |
| cg01490258 | X | | *BCOR* | 5'UTR | N Shore | 13.2 ± 2.9 | | 10.8 ± 1.9 | -2,4 | 1.38x10^-06^ | 0.102 | -0.418±0.077 | -0.463±0.088 |
| cg05026884 | X | | *BCOR* | 5'UTR | CpG Island | 26.5 ± 11.1 | | 19.4 ± 6.5 | -7,1 | 1.81x10^-04^ | 0.181 | -0.52±0.11 | -0.49±0.127 |
| cg10055320 | X | | *BCOR* | 5'UTR | N Shore | 42.2 ± 4.4 | | 37.5 ± 6.0 | -4,8 | 5.42x10^-05^ | 0.146 | -0.391±0.077 | -0.38±0.089 |
| cg15314890 | X | | *BCOR* | 5'UTR | CpG Island | 9.8 ± 7.6 | | 6.0 ± 4.0 | -3,8 | 3.20x10^-04^ | 0.193 | -0.486±0.109 | -0.466±0.126 |
| cg23701759 | X | | *BCOR* | 5'UTR | CpG Island | 34.9 ± 4.9 | | 30.3 ± 4.3 | -4,6 | 2.12x10^-04^ | 0.183 | -0.38±0.086 | -0.384±0.1 |
| cg24254387 | 1 | | *BTBD19;TCTEX1D4* | TSS1500 | S Shore | 9.3 ± 1.4 | | 8.0 ± 1.1 | -1,2 | 5.22x10^-04^ | 0.203 | -0.276±0.06 | -0.252±0.069 |
| cg10750182 | 10 | | *C10orf105;CDH23* | 5'UTR;1stExon;Body | Open sea | 56.0 ± 2.6 | | 53.0 ± 2.5 | -3 | 3.57x10^-03^ | 0.278 | -0.196±0.042 | -0.148±0.047 |
| cg27366964 | 10 | | *C10orf11* | Body | Open sea | 76.6 ± 3.5 | | 80.3 ± 3.1 | 3,7 | 3.42x10^-03^ | 0.275 | 0.357±0.079 | 0.261±0.087 |
| cg10611016 | 11 | | *C11orf42* | TSS1500 | Open sea | 44.9 ± 5.4 | | 38.1 ± 6.4 | -6,8 | 1.47x10^-04^ | 0.179 | -0.457±0.105 | -0.476±0.121 |
| cg15007983 | 19 | | *C19orf60* | TSS200 | N Shore | 20.3 ± 7.9 | | 14.6 ± 6.6 | -5,7 | 2.82x10^-05^ | 0.135 | -0.914±0.196 | -0.982±0.225 |
| cg15428479 | 19 | | *C19orf60* | TSS1500 | N Shore | 40.8 ± 5.7 | | 34.6 ± 5.3 | -6,3 | 1.34x10^-06^ | 0.102 | -0.495±0.092 | -0.551±0.105 |
| cg22038059 | 19 | | *C19orf60* | TSS200 | N Shore | 18.1 ± 3.7 | | 15.0 ± 3.3 | -3,1 | 1.15x10^-05^ | 0.123 | -0.456±0.097 | -0.517±0.111 |
| cg25234359 | 19 | | *C19orf60* | TSS1500 | N Shore | 55.7 ± 7.5 | | 50.6 ± 6.9 | -5,2 | 1.32x10^-04^ | 0.179 | -0.47±0.108 | -0.492±0.124 |
| cg00507154 | 19 | | *C19orf76* | 1stExon;5'UTR | CpG Island | 70.2 ± 4.6 | | 66.6 ± 4.0 | -3,6 | 5.29x10^-03^ | 0.306 | -0.339±0.076 | -0.235±0.083 |
| cg09358422 | 1 | | *C1orf159* | Body | CpG Island | 96.1 ± 0.7 | | 95.6 ± 0.6 | -0,5 | 1.20x10^-04^ | 0.176 | -0.305±0.066 | -0.31±0.076 |
| cg14989202 | 1 | | *C1orf200;PIK3CD* | TSS200;5'UTR | S Shore | 18.0 ± 2.5 | | 15.5 ± 2.4 | -2,5 | 5.33x10^-04^ | 0.203 | -0.289±0.061 | -0.252±0.069 |
| cg09563216 | 1 | | *C1orf51* | 1stExon;5'UTR | S Shore | 49.3 ± 4.1 | | 45.8 ± 4.1 | -3,5 | 3.47x10^-04^ | 0.196 | -0.253±0.053 | -0.231±0.061 |
| cg18157896 | 20 | | *C20orf141;LOC100288797* | TSS200;TSS1500 | Open sea | 38.3 ± 3.2 | | 35.1 ± 3.6 | -3,2 | 2.48x10^-04^ | 0.186 | -0.256±0.055 | -0.25±0.064 |
| cg21183455 | 22 | | *C22orf34* | TSS1500 | Open sea | 66.4 ± 5.8 | | 60.4 ± 5.7 | -6 | 5.22x10^-04^ | 0.203 | -0.445±0.102 | -0.42±0.118 |
| cg14580211 | 5 | | *C5orf62* | Body | S Shelf | 69.3 ± 3.4 | | 64.5 ± 5.0 | -4,8 | 1.44x10^-03^ | 0.238 | -0.339±0.073 | -0.267±0.081 |
| cg23599776 | 6 | | *C6orf134* | Body | N Shelf | 80.9 ± 2.2 | | 78.8 ± 2.1 | -2 | 4.13x10^-04^ | 0.202 | -0.229±0.05 | -0.219±0.058 |
| cg01048372 | 8 | | *C8orf73* | TSS1500 | S Shore | 43.8 ± 3.8 | | 39.0 ± 4.1 | -4,8 | 4.40x10^-04^ | 0.202 | -0.314±0.069 | -0.293±0.08 |
| cg08601628 | 8 | | *C8orf74* | Body | Open sea | 82.5 ± 3.0 | | 78.4 ± 4.2 | -4,1 | 3.33x10^-04^ | 0.195 | -0.429±0.09 | -0.383±0.103 |
| cg14059835 | 9 | | *C9orf78;USP20* | TSS1500;5'UTR | S Shore | 13.0 ± 1.8 | | 11.0 ± 1.8 | -2 | 6.32x10^-05^ | 0.157 | -0.327±0.073 | -0.355±0.084 |
| cg04334723 | 19 | | *CALR* | Body | N Shelf | 40.3 ± 3.8 | | 35.7 ± 4.8 | -4,6 | 1.24x10^-04^ | 0.178 | -0.334±0.073 | -0.341±0.084 |
| cg17118262 | 17 | | *CCL1* | TSS1500 | Open sea | 28.5 ± 3.6 | | 23.9 ± 4.0 | -4,5 | 4.63x10^-05^ | 0.145 | -0.386±0.074 | -0.367±0.085 |
| cg04780563 | 10 | | *CCNY* | Body;5'UTR | Open sea | 69.6 ± 4.2 | | 73.3 ± 3.3 | 3,7 | 3.06x10^-04^ | 0.193 | 0.317±0.067 | 0.293±0.077 |
| cg15518113 | 1 | | *CD247* | 3'UTR | Open sea | 79.1 ± 3.0 | | 82.5 ± 2.9 | 3,4 | 1.32x10^-05^ | 0.123 | 0.378±0.078 | 0.414±0.089 |
| cg17215278 | 1 | | *CD58* | TSS1500 | S Shore | 50.9 ± 4.0 | | 47.0 ± 3.9 | -4 | 7.72x10^-04^ | 0.217 | -0.269±0.059 | -0.238±0.067 |
| cg04057956 | 12 | | *CD9* | Body | Open sea | 58.9 ± 4.0 | | 54.6 ± 3.8 | -4,4 | 1.36x10^-04^ | 0.179 | -0.283±0.061 | -0.287±0.071 |
| cg11811510 | 19 | | *CEACAM1* | TSS200 | Open sea | 10.9 ± 1.6 | | 9.0 ± 1.3 | -1,9 | 6.91x10^-05^ | 0.160 | -0.294±0.06 | -0.297±0.07 |
| cg13134650 | 1 | | *CHI3L1* | TSS1500 | Open sea | 75.2 ± 2.6 | | 72.1 ± 2.7 | -3,1 | 1.83x10^-05^ | 0.131 | -0.268±0.052 | -0.281±0.06 |
| cg15030712 | 7 | | *CHN2* | Body | Open sea | 30.5 ± 2.6 | | 26.6 ± 4.5 | -3,8 | 3.25x10^-05^ | 0.137 | -0.319±0.07 | -0.354±0.08 |
| cg00254556 | 8 | | *CHRNA2* | 3'UTR | N Shore | 67.9 ± 1.5 | | 65.2 ± 2.5 | -2,6 | 4.35x10^-05^ | 0.145 | -0.184±0.038 | -0.20±0.043 |
| cg02235659 | 3 | | *CLEC3B* | TSS1500 | Open sea | 48.9 ± 3.1 | | 44.8 ± 4.2 | -4,1 | 6.95x10^-06^ | 0.112 | -0.278±0.052 | -0.298±0.06 |
| cg10521431 | 10 | | *CNNM2* | Body | Open sea | 85.4 ± 1.7 | | 87.4 ± 1.4 | 2 | 7.20x10^-05^ | 0.160 | 0.29±0.054 | 0.264±0.062 |
| cg13917614 | 17 | | *CNP* | Body | Open sea | 79.6 ± 4.2 | | 84.2 ± 3.7 | 4,7 | 2.07x10^-05^ | 0.131 | 0.553±0.106 | 0.549±0.123 |
| cg01055562 | 1 | | *COG2* | TSS1500 | N Shore | 49.2 ± 3.2 | | 45.8 ± 3.1 | -3,4 | 9.83x10^-05^ | 0.171 | -0.232±0.047 | -0.231±0.054 |
| cg02266731 | 12 | | *CPM* | TSS1500 | Open sea | 33.3 ± 3.3 | | 28.6 ± 4.6 | -4,7 | 6.23x10^-04^ | 0.210 | -0.347±0.077 | -0.314±0.089 |
| cg12678834 | 11 | | *CXCR5* | TSS1500 | Open sea | 70.2 ± 1.9 | | 67.8 ± 2.0 | -2,4 | 9.90x10^-04^ | 0.226 | -0.18±0.038 | -0.158±0.044 |
| cg27067618 | 19 | | *CYP4F3* | 5'UTR | Open sea | 26.8 ± 2.9 | | 23.1 ± 3.8 | -3,7 | 3.97x10^-05^ | 0.145 | -0.333±0.066 | -0.334±0.076 |
| cg24690709 | 4 | | *DAPP1* | Body | Open sea | 17.2 ± 1.6 | | 15.1 ± 2.2 | -2 | 1.91x10^-04^ | 0.181 | -0.27±0.056 | -0.256±0.064 |
| cg10679266 | 8 | | *DEFA4* | TSS200 | Open sea | 56.1 ± 4.6 | | 50.8 ± 4.9 | -5,3 | 2.28x10^-04^ | 0.185 | -0.354±0.079 | -0.352±0.092 |
| cg05624376 | 2 | | *DHRS9* | Body | Open sea | 45.2 ± 4.6 | | 38.6 ± 4.8 | -6,6 | 5.44x10^-05^ | 0.146 | -0.429±0.082 | -0.403±0.095 |
| cg12993916 | 6 | | *DTNBP1* | Body | Open sea | 78.9 ± 3.0 | | 76.4 ± 3.2 | -2,4 | 2.13x10^-04^ | 0.183 | -0.291±0.065 | -0.293±0.075 |
| cg07019857 | 5 | | *EFNA5* | Body | Open sea | 53.0 ± 3.1 | | 48.3 ± 3.7 | -4,6 | 5.01x10^-05^ | 0.145 | -0.301±0.056 | -0.282±0.064 |
| cg23060465 | 8 | | *EIF2C2* | Body | Open sea | 92.6 ± 1.9 | | 94.4 ± 1.2 | 1,8 | 2.70x10^-04^ | 0.187 | 0.453±0.103 | 0.445±0.119 |
| cg00431050 | 10 | | *ELOVL3* | TSS1500 | N Shore | 60.9 ± 2.6 | | 57.9 ± 2.4 | -3 | 2.26x10^-04^ | 0.185 | -0.207±0.042 | -0.196±0.048 |
| cg00262446 | 4 | | *EMCN* | 5'UTR;1stExon | Open sea | 18.0 ± 1.9 | | 15.5 ± 2.1 | -2,5 | 3.67x10^-04^ | 0.196 | -0.291±0.062 | -0.266±0.071 |
| cg14041338 | 13 | | *ENOX1* | Body | Open sea | 58.6 ± 4.2 | | 52.8 ± 6.0 | -5,8 | 3.09x10^-05^ | 0.137 | -0.399±0.084 | -0.424±0.096 |
| cg11147155 | 1 | | *ENSA* | 3'UTR | Open sea | 39.6 ± 3.8 | | 34.7 ± 4.5 | -4,9 | 1.15x10^-04^ | 0.176 | -0.337±0.075 | -0.351±0.087 |
| cg10539861 | 10 | | *ENTPD7* | TSS1500 | N Shore | 12.2 ± 2.8 | | 10.2 ± 2.5 | -2 | 6.88x10^-04^ | 0.213 | -0.435±0.096 | -0.383±0.11 |
| cg10506318 | 5 | | *ERCC8;NDUFAF2* | Body;TSS1500 | N Shore | 39.0 ± 3.7 | | 33.6 ± 5.0 | -5,4 | 2.77x10^-04^ | 0.188 | -0.365±0.081 | -0.353±0.094 |
| cg03636183 | 19 | | *F2RL3* | Body | N Shore | 68.9 ± 4.4 | | 63.0 ± 7.1 | -5,9 | 4.84x10^-03^ | 0.301 | -0.449±0.099 | -0.305±0.107 |
| cg02708705 | 17 | | *FAM100B* | Body | S Shelf | 17.6 ± 2.7 | | 14.9 ± 2.5 | -2,7 | 2.64x10^-04^ | 0.187 | -0.335±0.072 | -0.316±0.083 |
| cg15259233 | 6 | | *FAM120B* | Body | Open sea | 84.9 ± 2.0 | | 87.0 ± 1.4 | 2,2 | 6.12x10^-04^ | 0.210 | 0.268±0.056 | 0.233±0.064 |
| cg10206969 | 7 | | *FBXL18* | 3'UTR | N Shelf | 21.8 ± 1.8 | | 19.6 ± 2.1 | -2,1 | 1.29x10^-04^ | 0.179 | -0.24±0.048 | -0.229±0.055 |
| cg05890727 | 14 | | *FBXO34* | 5'UTR | N Shore | 50.0 ± 4.3 | | 44.6 ± 4.4 | -5,4 | 8.86x10^-05^ | 0.167 | -0.341±0.074 | -0.352±0.085 |
| cg01383287 | 2 | | *FBXO41* | Body | N Shore | 79.3 ± 3.1 | | 76.1 ± 2.8 | -3,2 | 2.62x10^-04^ | 0.187 | -0.324±0.065 | -0.284±0.074 |
| cg10220544 | 1 | | *FGGY* | Body | Open sea | 36.4 ± 4.7 | | 30.7 ± 5.0 | -5,7 | 5.42x10^-05^ | 0.146 | -0.42±0.088 | -0.432±0.102 |
| cg21199922 | 4 | | *FLJ13197* | Body | Open sea | 35.9 ± 3.7 | | 30.3 ± 5.2 | -5,6 | 5.16x10^-05^ | 0.145 | -0.405±0.083 | -0.411±0.096 |
| cg22791543 | 5 | | *FLJ33630* | Body | N Shelf | 81.1 ± 2.0 | | 78.6 ± 2.0 | -2,5 | 1.62x10^-03^ | 0.241 | -0.229±0.051 | -0.193±0.058 |
| cg05053979 | 14 | | *FOXN3* | Body | Open sea | 66.5 ± 4.5 | | 62.3 ± 5.0 | -4,1 | 1.13x10^-03^ | 0.228 | -0.328±0.074 | -0.282±0.084 |
| cg12688265 | 1 | | *GBAP1* | TSS1500 | S Shore | 10.2 ± 1.2 | | 8.9 ± 1.0 | -1,3 | 5.01x10^-05^ | 0.145 | -0.251±0.052 | -0.265±0.06 |
| cg04574552 | X | | *GEMIN8* | 5'UTR | N Shore | 10.5 ± 4.6 | | 8.0 ± 2.6 | -2,5 | 8.37x10^-04^ | 0.219 | -0.36±0.08 | -0.313±0.091 |
| cg09674502 | 1 | | *GFI1* | TSS1500 | S Shore | 49.5 ± 3.3 | | 43.5 ± 5.1 | -6,1 | 1.61x10^-05^ | 0.129 | -0.388±0.072 | -0.385±0.083 |
| cg25320328 | 1 | | *GFI1* | TSS1500 | S Shore | 35.4 ± 3.6 | | 30.7 ± 4.7 | -4,6 | 4.82x10^-05^ | 0.145 | -0.35±0.077 | -0.381±0.089 |
| cg14457284 | 9 | | *GFI1B* | TSS1500 | Open sea | 29.5 ± 3.1 | | 25.9 ± 3.0 | -3,6 | 3.55x10^-04^ | 0.196 | -0.284±0.061 | -0.265±0.071 |
| cg02942825 | 19 | | *GIPR* | 3'UTR | S Shore | 49.8 ± 3.2 | | 46.5 ± 3.2 | -3,3 | 2.28x10^-06^ | 0.102 | -0.234±0.05 | -0.294±0.055 |
| cg17808910 | 7 | | *GLCCI1* | Body | S Shelf | 73.7 ± 3.9 | | 77.5 ± 2.9 | 3,8 | 2.07x10^-05^ | 0.131 | 0.372±0.072 | 0.381±0.084 |
| cg21380380 | 7 | | *GLCCI1* | Body | S Shelf | 63.1 ± 5.2 | | 68.7 ± 3.4 | 5,5 | 1.12x10^-04^ | 0.175 | 0.367±0.074 | 0.344±0.085 |
| cg26567012 | 5 | | *GLRA1* | 3'UTR | Open sea | 55.4 ± 3.8 | | 48.8 ± 5.2 | -6,5 | 1.67x10^-06^ | 0.102 | -0.399±0.077 | -0.457±0.088 |
| cg05551922 | 3 | | *GPX1* | TSS1500 | S Shore | 10.7 ± 2.2 | | 8.6 ± 1.4 | -2,1 | 3.17x10^-05^ | 0.137 | -0.379±0.08 | -0.405±0.092 |
| cg22312904 | 8 | | *GSDMD* | TSS1500;5'UTR | N Shore | 46.6 ± 3.5 | | 42.1 ± 4.0 | -4,5 | 2.89x10^-04^ | 0.190 | -0.29±0.064 | -0.282±0.074 |
| cg21205139 | 4 | | *GUCY1B3* | Body | S Shore | 26.6 ± 2.2 | | 23.3 ± 2.7 | -3,2 | 5.11x10^-05^ | 0.145 | -0.268±0.054 | -0.272±0.062 |
| cg08766149 | 14 | | *GZMB* | Body | Open sea | 77.5 ± 3.6 | | 81.1 ± 2.3 | 3,6 | 1.62x10^-05^ | 0.129 | 0.371±0.07 | 0.374±0.081 |
| cg05903736 | 2 | | *HDAC4* | Body | S Shore | 20.8 ± 3.2 | | 17.9 ± 4.1 | -2,9 | 1.34x10^-04^ | 0.179 | -0.374±0.084 | -0.388±0.097 |
| cg06117093 | 2 | | *HDAC4* | 5'UTR | Open sea | 22.7 ± 4.0 | | 19.0 ± 3.1 | -3,8 | 5.61x10^-04^ | 0.205 | -0.383±0.086 | -0.35±0.098 |
| cg07554496 | 2 | | *HDAC4* | Body | Open sea | 87.5 ± 2.6 | | 89.5 ± 1.3 | 2 | 3.91x10^-04^ | 0.200 | 0.335±0.077 | 0.325±0.088 |
| cg15978561 | 2 | | *HDAC4* | Body | CpG Island | 14.5 ± 3.0 | | 12.0 ± 2.7 | -2,5 | 1.42x10^-05^ | 0.126 | -0.419±0.078 | -0.419±0.091 |
| cg01088404 | 12 | | *HDAC7* | TSS1500 | S Shore | 34.0 ± 3.7 | | 29.7 ± 4.0 | -4,3 | 1.11x10^-03^ | 0.227 | -0.33±0.074 | -0.285±0.085 |
| cg16659880 | 15 | | *HERC1* | Body | Open sea | 90.4 ± 1.1 | | 89.1 ± 1.4 | -1,3 | 3.19x10^-05^ | 0.137 | -0.253±0.053 | -0.276±0.061 |
| cg20294304 | 6 | | *HMGA1* | TSS1500 | CpG Island | 7.5 ± 3.4 | | 5.6 ± 1.2 | -1,9 | 4.84x10^-05^ | 0.145 | -0.479±0.104 | -0.51±0.12 |
| cg23867647 | 19 | | *HSPB6* | Body | CpG Island | 66.6 ± 5.0 | | 62.5 ± 4.2 | -4,1 | 2.89x10^-04^ | 0.190 | -0.316±0.068 | -0.3±0.079 |
| cg09494176 | 4 | | *HTT* | Body | Open sea | 84.6 ± 2.2 | | 87.1 ± 2.0 | 2,5 | 8.49x10^-05^ | 0.167 | 0.343±0.068 | 0.327±0.079 |
| cg07271561 | 3 | | *HYAL2* | 5'UTR;TSS1500 | CpG Island | 15.5 ± 1.7 | | 13.2 ± 1.7 | -2,2 | 1.41x10^-04^ | 0.179 | -0.265±0.059 | -0.277±0.068 |
| cg13341668 | 3 | | *HYAL2* | 5'UTR;TSS1500 | CpG Island | 84.4 ± 3.3 | | 81.5 ± 2.5 | -2,9 | 4.81x10^-05^ | 0.145 | -0.369±0.073 | -0.365±0.085 |
| cg13298466 | 12 | | *IFFO1* | Body;1stExon;5'UTR | S Shore | 50.5 ± 3.9 | | 44.4 ± 5.5 | -6,1 | 2.70x10^-04^ | 0.187 | -0.364±0.082 | -0.359±0.095 |
| cg18612209 | 10 | | *INPP5A* | Body | Open sea | 82.0 ± 2.6 | | 84.5 ± 1.9 | 2,5 | 9.33x10^-05^ | 0.169 | 0.314±0.062 | 0.295±0.071 |
| cg14042143 | 7 | | *IQCE* | Body | S Shore | 70.7 ± 5.5 | | 65.5 ± 5.6 | -5,2 | 6.28x10^-06^ | 0.109 | -0.465±0.08 | -0.445±0.092 |
| cg16313758 | 7 | | *IQCE* | Body | S Shore | 65.2 ± 4.2 | | 61.0 ± 4.7 | -4,2 | 8.13x10^-05^ | 0.167 | -0.319±0.059 | -0.287±0.068 |
| cg18338984 | 7 | | *IQCE* | Body | S Shore | 52.9 ± 5.4 | | 48.6 ± 5.2 | -4,3 | 1.09x10^-04^ | 0.175 | -0.318±0.067 | -0.315±0.077 |
| cg17516539 | 5 | | *ITK* | 3'UTR | Open sea | 29.0 ± 4.6 | | 24.1 ± 4.3 | -4,9 | 1.13x10^-05^ | 0.123 | -0.445±0.091 | -0.485±0.104 |
| cg17683336 | 19 | | *KANK2* | 5'UTR;TSS1500 | N Shore | 10.5 ± 1.3 | | 8.9 ± 1.1 | -1,6 | 7.13x10^-05^ | 0.160 | -0.262±0.058 | -0.284±0.067 |
| cg15153383 | 16 | | *KATNB1* | 5'UTR | S Shore | 9.5 ± 1.2 | | 8.5 ± 0.5 | -1 | 2.54x10^-05^ | 0.135 | -0.201±0.042 | -0.227±0.048 |
| cg25199552 | 1 | | *KDM1A* | Body | S Shelf | 54.2 ± 5.0 | | 47.0 ± 6.3 | -7,2 | 1.74x10^-05^ | 0.131 | -0.472±0.096 | -0.5±0.11 |
| cg17737314 | 1 | | *KDM4A* | TSS1500 | N Shore | 37.1 ± 5.0 | | 30.7 ± 5.8 | -6,5 | 1.84x10^-04^ | 0.181 | -0.458±0.096 | -0.428±0.11 |
| cg15206171 | 6 | | *KIFC1* | TSS1500 | N Shore | 46.2 ± 3.9 | | 41.1 ± 4.9 | -5,1 | 4.36x10^-04^ | 0.202 | -0.331±0.076 | -0.318±0.087 |
| cg08326410 | 19 | | *KIR2DL4* | TSS200 | Open sea | 83.4 ± 2.3 | | 86.4 ± 1.9 | 3 | 8.47x10^-06^ | 0.121 | 0.405±0.065 | 0.355±0.073 |
| cg04339360 | 13 | | *KLF5* | Body | S Shore | 48.7 ± 4.2 | | 42.0 ± 5.8 | -6,6 | 3.09x10^-05^ | 0.137 | -0.444±0.086 | -0.439±0.1 |
| cg15956469 | 12 | | *KLRD1* | Body | Open sea | 86.8 ± 3.1 | | 89.9 ± 1.8 | 3,1 | 2.16x10^-05^ | 0.131 | 0.483±0.094 | 0.485±0.108 |
| cg15500907 | 6 | | *LAMA4* | Body | Open sea | 38.5 ± 5.7 | | 31.9 ± 4.7 | -6,7 | 6.83x10^-05^ | 0.160 | -0.404±0.092 | -0.439±0.105 |
| cg17893934 | 10 | | *LARP4B* | 3'UTR | S Shelf | 92.1 ± 1.5 | | 93.6 ± 1.1 | 1,5 | 2.40x10^-03^ | 0.257 | 0.341±0.075 | 0.26±0.084 |
| cg16183122 | 10 | | *LDB3* | Body | Open sea | 92.2 ± 1.6 | | 93.7 ± 1.1 | 1,6 | 2.97x10^-04^ | 0.191 | 0.369±0.083 | 0.36±0.096 |
| cg20759281 | 12 | | *LDHB* | Body | Open sea | 29.4 ± 3.7 | | 25.4 ± 4.0 | -4,1 | 1.96x10^-04^ | 0.181 | -0.336±0.076 | -0.341±0.088 |
| cg11247817 | 5 | | *LNPEP* | Body;5'UTR;1stExon | Open sea | 39.3 ± 4.3 | | 34.1 ± 4.2 | -5,3 | 1.17x10^-03^ | 0.229 | -0.355±0.078 | -0.294±0.088 |
| cg23484268 | 15 | | *LOXL1* | Body | S Shore | 72.1 ± 2.8 | | 69.0 ± 3.8 | -3,1 | 4.71x10^-05^ | 0.145 | -0.285±0.062 | -0.31±0.071 |
| cg15052335 | 18 | | *LPIN2* | 5'UTR;1stExon | N Shore | 71.7 ± 3.5 | | 75.2 ± 2.5 | 3,5 | 8.35x10^-04^ | 0.219 | 0.282±0.063 | 0.254±0.073 |
| cg00902153 | 3 | | *LPP* | Body | Open sea | 22.2 ± 2.6 | | 19.4 ± 3.4 | -2,8 | 4.08x10^-05^ | 0.145 | -0.317±0.072 | -0.36±0.083 |
| cg21668832 | 6 | | *LRRC16A* | TSS1500 | N Shore | 24.1 ± 4.2 | | 20.1 ± 3.8 | -3,9 | 5.17x10^-05^ | 0.145 | -0.425±0.089 | -0.435±0.102 |
| cg11918450 | 2 | | *LTBP1* | Body;TSS1500 | Open sea | 47.4 ± 3.8 | | 41.5 ± 5.0 | -5,9 | 8.55x10^-05^ | 0.167 | -0.358±0.074 | -0.356±0.086 |
| cg25136988 | 6 | | *LY6G5C* | TSS1500 | N Shore | 67.5 ± 4.2 | | 64.2 ± 3.4 | -3,3 | 3.63x10^-04^ | 0.196 | -0.302±0.065 | -0.282±0.075 |
| cg24760467 | 10 | | *LZTS2* | 5'UTR | S Shore | 49.5 ± 3.7 | | 44.8 ± 4.6 | -4,8 | 5.44x10^-05^ | 0.146 | -0.316±0.071 | -0.347±0.081 |
| cg23015664 | 7 | | *MAD1L1* | Body | Open sea | 88.6 ± 2.3 | | 90.9 ± 1.5 | 2,3 | 2.81x10^-05^ | 0.135 | 0.408±0.081 | 0.416±0.094 |
| cg15521790 | 11 | | *MAML2* | Body | Open sea | 48.4 ± 3.4 | | 44.6 ± 4.1 | -3,8 | 2.73x10^-04^ | 0.187 | -0.269±0.058 | -0.257±0.067 |
| cg06706159 | 19 | | *MAST3* | Body | CpG Island | 83.0 ± 5.9 | | 89.4 ± 4.4 | 6,4 | 6.41x10^-05^ | 0.157 | 0.897±0.188 | 0.895±0.217 |
| cg24137511 | 19 | | *MAST3* | Body | CpG Island | 86.4 ± 4.6 | | 90.9 ± 3.0 | 4,5 | 1.48x10^-04^ | 0.179 | 0.713±0.149 | 0.669±0.171 |
| cg16537483 | 3 | | *MBNL1* | Body | Open sea | 59.1 ± 2.8 | | 55.0 ± 3.4 | -4,1 | 3.37x10^-05^ | 0.139 | -0.248±0.053 | -0.274±0.061 |
| cg20351875 | 12 | | *MIR548C;RASSF3* | TSS1500;Body | Open sea | 44.1 ± 4.3 | | 38.4 ± 4.9 | -5,7 | 2.81x10^-04^ | 0.189 | -0.362±0.079 | -0.345±0.091 |
| cg11355029 | 19 | | *MLLT1* | Body | N Shelf | 51.8 ± 2.5 | | 48.7 ± 2.9 | -3,2 | 2.13x10^-04^ | 0.183 | -0.22±0.044 | -0.206±0.051 |
| cg05651778 | 17 | | *MRC2* | Body | CpG Island | 5.8 ± 1.6 | | 4.7 ± 0.8 | -1,1 | 2.60x10^-04^ | 0.187 | -0.322±0.072 | -0.317±0.083 |
| cg10956549 | 11 | | *MTL5* | 3'UTR;Body | Open sea | 87.2 ± 1.1 | | 85.5 ± 0.9 | -1,7 | 5.64x10^-05^ | 0.147 | -0.193±0.036 | -0.19±0.041 |
| cg14175330 | 9 | | *NACC2* | 5'UTR | Open sea | 68.7 ± 3.9 | | 64.4 ± 3.8 | -4,3 | 1.69x10^-04^ | 0.181 | -0.337±0.072 | -0.33±0.084 |
| cg08869244 | 11 | | *NDUFV1* | TSS1500 | N Shore | 49.2 ± 2.1 | | 45.9 ± 2.6 | -3,2 | 1.32x10^-05^ | 0.123 | -0.207±0.038 | -0.218±0.044 |
| cg01010839 | 10 | | *NET1* | TSS1500;Body | N Shore | 71.2 ± 4.3 | | 67.7 ± 3.0 | -3,5 | 4.10x10^-06^ | 0.109 | -0.283±0.061 | -0.345±0.068 |
| cg25145459 | 18 | | *NFATC1* | Body;TSS200;5'UTR | CpG Island | 2.8 ± 0.5 | | 3.4 ± 0.6 | 0,6 | 4.10x10^-05^ | 0.145 | 0.338±0.072 | 0.362±0.083 |
| cg04158018 | 12 | | *NFE2* | TSS1500 | Open sea | 31.4 ± 3.4 | | 28.3 ± 3.2 | -3,1 | 4.30x10^-03^ | 0.291 | -0.258±0.056 | -0.184±0.062 |
| cg23140706 | 12 | | *NFE2* | 5'UTR | Open sea | 46.0 ± 2.3 | | 42.8 ± 3.1 | -3,2 | 1.39x10^-04^ | 0.179 | -0.223±0.043 | -0.206±0.049 |
| cg24925865 | 7 | | *NOM1* | 1stExon | CpG Island | 6.6 ± 0.7 | | 7.5 ± 1.0 | 0,9 | 6.57x10^-06^ | 0.109 | 0.236±0.048 | 0.274±0.054 |
| cg12613344 | 11 | | *NR1H3* | 5'UTR | Open sea | 63.4 ± 3.5 | | 59.3 ± 3.7 | -4,1 | 1.21x10^-03^ | 0.230 | -0.259±0.058 | -0.225±0.066 |
| cg27122888 | 11 | | *NRXN2* | Body | S Shelf | 14.9 ± 1.8 | | 12.3 ± 1.7 | -2,6 | 2.71x10^-05^ | 0.135 | -0.333±0.06 | -0.313±0.069 |
| cg27209729 | 11 | | *NRXN2* | Body | S Shore | 56.5 ± 5.8 | | 51.7 ± 5.9 | -4,8 | 2.69x10^-05^ | 0.135 | -0.347±0.074 | -0.38±0.085 |
| cg04992150 | 3 | | *NUP210* | Body | N Shelf | 28.9 ± 2.7 | | 25.1 ± 3.4 | -3,8 | 9.79x10^-05^ | 0.171 | -0.321±0.067 | -0.317±0.077 |
| cg20109495 | 17 | | *OR3A2* | TSS1500 | Open sea | 78.1 ± 3.3 | | 74.2 ± 3.7 | -3,8 | 1.90x10^-03^ | 0.249 | -0.36±0.078 | -0.279±0.088 |
| cg12892799 | 3 | | *OXSR1* | 1stExon;5'UTR | CpG Island | 10.3 ± 16.1 | | 5.6 ± 1.4 | -4,6 | 6.71x10^-05^ | 0.160 | -0.974±0.20 | -0.95±0.231 |
| cg04685387 | 10 | | *PARD3* | Body | Open sea | 50.5 ± 3.9 | | 47.0 ± 3.2 | -3,5 | 8.07x10^-05^ | 0.167 | -0.259±0.056 | -0.272±0.064 |
| cg09080522 | 22 | | *PARVG* | TSS1500 | Open sea | 26.1 ± 2.9 | | 22.3 ± 3.3 | -3,7 | 2.05x10^-04^ | 0.182 | -0.323±0.073 | -0.326±0.084 |
| cg10117603 | 13 | | *PCCA* | Body | Open sea | 87.2 ± 1.6 | | 88.9 ± 1.5 | 1,7 | 1.47x10^-04^ | 0.179 | 0.293±0.056 | 0.261±0.064 |
| cg15559940 | 13 | | *PCCA* | Body | Open sea | 87.2 ± 1.7 | | 89.1 ± 0.9 | 1,9 | 9.11x10^-05^ | 0.167 | 0.285±0.052 | 0.253±0.06 |
| cg00908004 | 13 | | *PCID2* | Body | Open sea | 81.7 ± 1.8 | | 83.8 ± 1.9 | 2,1 | 4.45x10^-04^ | 0.202 | 0.24±0.052 | 0.224±0.06 |
| cg06567722 | 13 | | *PCID2* | Body | Open sea | 93.3 ± 2.1 | | 95.5 ± 1.2 | 2,3 | 6.87x10^-04^ | 0.213 | 0.543±0.123 | 0.489±0.142 |
| cg06619077 | 1 | | *PDZK1IP1* | TSS1500 | Open sea | 52.9 ± 4.7 | | 48.6 ± 5.3 | -4,3 | 4.30x10^-05^ | 0.145 | -0.335±0.071 | -0.356±0.082 |
| cg05694563 | 17 | | *PEMT* | Body | CpG Island | 43.0 ± 2.7 | | 39.7 ± 3.5 | -3,2 | 3.63x10^-05^ | 0.140 | -0.237±0.053 | -0.27±0.06 |
| cg11978441 | 1 | | *PER3* | Body | Open sea | 77.6 ± 2.4 | | 80.7 ± 2.0 | 3,1 | 4.19x10^-05^ | 0.145 | 0.308±0.054 | 0.273±0.061 |
| cg27545615 | 10 | | *PFKFB3* | Body | S Shelf | 39.4 ± 4.7 | | 33.5 ± 4.9 | -5,9 | 2.73x10^-04^ | 0.187 | -0.411±0.09 | -0.391±0.104 |
| cg11940177 | 10 | | *PGAM1* | Body | S Shelf | 28.2 ± 3.4 | | 24.1 ± 4.3 | -4 | 6.35x10^-04^ | 0.210 | -0.333±0.075 | -0.306±0.086 |
| cg09980384 | 17 | | *PGS1* | Body | Open sea | 12.6 ± 1.7 | | 10.6 ± 1.6 | -2 | 1.12x10^-04^ | 0.175 | -0.332±0.066 | -0.309±0.076 |
| cg10517290 | 17 | | *PGS1* | Body | S Shelf | 11.0 ± 1.4 | | 9.7 ± 1.2 | -1,3 | 2.93x10^-04^ | 0.191 | -0.237±0.05 | -0.225±0.058 |
| cg24796663 | 6 | | *PHF1* | TSS1500 | N Shore | 12.9 ± 1.8 | | 11.4 ± 1.2 | -1,6 | 3.73x10^-04^ | 0.197 | -0.255±0.054 | -0.235±0.062 |
| cg07730673 | 3 | | *PIGX;C3orf34* | TSS1500;5'UTR | N Shore | 9.3 ± 0.7 | | 8.3 ± 0.7 | -1 | 1.41x10^-04^ | 0.179 | -0.18±0.038 | -0.186±0.043 |
| cg22234930 | 15 | | *PKM2* | 5'UTR | N Shelf | 12.6 ± 1.6 | | 10.7 ± 1.7 | -1,8 | 4.10x10^-04^ | 0.201 | -0.292±0.06 | -0.257±0.069 |
| cg03792042 | 8 | | *PLEC1* | Body | N Shelf | 58.9 ± 2.6 | | 53.8 ± 6.5 | -5,1 | 1.63x10^-03^ | 0.242 | -0.355±0.08 | -0.295±0.091 |
| cg11224624 | 8 | | *PLEC1* | Body | CpG Island | 2.1 ± 0.3 | | 2.4 ± 0.3 | 0,3 | 3.28x10^-05^ | 0.137 | 0.258±0.056 | 0.289±0.064 |
| cg23098529 | 19 | | *PPAN;PPAN-P2RY11* | TSS1500 | N Shore | 28.1 ± 2.8 | | 24.1 ± 3.9 | -4 | 1.10x10^-04^ | 0.175 | -0.346±0.072 | -0.341±0.084 |
| cg05713693 | 4 | | *PRDM5* | 1stExon | CpG Island | 7.3 ± 0.6 | | 8.3 ± 0.8 | 1 | 1.87x10^-04^ | 0.181 | 0.224±0.041 | 0.192±0.046 |
| cg13775629 | 10 | | *PRF1* | Body | CpG Island | 74.2 ± 4.1 | | 77.6 ± 2.7 | 3,5 | 1.37x10^-04^ | 0.179 | 0.326±0.074 | 0.343±0.086 |
| cg02480298 | 11 | | *PRR5L* | 5'UTR;TSS1500;Body | Open sea | 83.6 ± 1.9 | | 85.7 ± 1.8 | 2,1 | 7.65x10^-03^ | 0.341 | 0.259±0.058 | 0.173±0.063 |
| cg23351584 | 11 | | *PRSS23* | 5'UTR | S Shore | 17.1 ± 1.6 | | 15.1 ± 1.7 | -2 | 5.96x10^-04^ | 0.208 | -0.231±0.049 | -0.207±0.056 |
| cg03065803 | 10 | | *PSAP* | 3'UTR | Open sea | 90.2 ± 1.2 | | 88.9 ± 1.3 | -1,3 | 3.32x10^-06^ | 0.109 | -0.248±0.048 | -0.286±0.055 |
| cg26197915 | 11 | | *PTPRJ* | Body | Open sea | 37.6 ± 3.6 | | 32.5 ± 5.1 | -5,1 | 1.14x10^-04^ | 0.176 | -0.37±0.083 | -0.384±0.095 |
| cg24514600 | 8 | | *PVT1* | TSS1500 | N Shore | 59.0 ± 5.1 | | 52.1 ± 5.9 | -6,8 | 1.40x10^-04^ | 0.179 | -0.427±0.096 | -0.436±0.11 |
| cg24175188 | 3 | | *PXK* | Body | Open sea | 52.1 ± 5.0 | | 46.3 ± 4.8 | -5,8 | 3.74x10^-04^ | 0.197 | -0.366±0.082 | -0.35±0.095 |
| cg18936471 | 4 | | *RAP1GDS1* | Body | S Shelf | 63.5 ± 3.8 | | 58.9 ± 3.3 | -4,6 | 2.32x10^-04^ | 0.185 | -0.294±0.064 | -0.288±0.074 |
| cg00054352 | 13 | | *RASA3* | Body | Open sea | 83.4 ± 3.5 | | 87.1 ± 2.6 | 3,7 | 1.84x10^-04^ | 0.181 | 0.46±0.094 | 0.421±0.109 |
| cg26181840 | 13 | | *RASA3* | Body | CpG Island | 82.7 ± 2.6 | | 85.9 ± 2.7 | 3,2 | 1.52x10^-05^ | 0.126 | 0.402±0.078 | 0.415±0.09 |
| cg00888521 | 5 | | *RASGRF2* | Body | CpG Island | 21.7 ± 10.7 | | 16.3 ± 2.8 | -5,3 | 6.52x10^-05^ | 0.158 | -0.605±0.138 | -0.658±0.159 |
| cg07043361 | 12 | | *RBM19* | TSS1500 | S Shore | 36.3 ± 3.8 | | 31.4 ± 4.6 | -4,8 | 1.01x10^-04^ | 0.172 | -0.341±0.077 | -0.362±0.089 |
| cg15636859 | 20 | | *RBM38* | Body;3'UTR | CpG Island | 78.2 ± 3.9 | | 82.1 ± 2.7 | 4 | 3.16x10^-05^ | 0.137 | 0.409±0.08 | 0.406±0.092 |
| cg13707794 | 21 | | *RCAN1* | 3'UTR | Open sea | 29.5 ± 3.5 | | 24.8 ± 4.3 | -4,7 | 6.37x10^-04^ | 0.210 | -0.353±0.08 | -0.323±0.092 |
| cg01447281 | 1 | | *RERE* | 5'UTR;Body | N Shore | 15.2 ± 2.2 | | 13.0 ± 2.7 | -2,2 | 6.28x10^-04^ | 0.210 | -0.346±0.076 | -0.309±0.087 |
| cg01112784 | 10 | | *RHOBTB1* | Body | Open sea | 76.9 ± 2.4 | | 79.1 ± 1.8 | 2,3 | 5.65x10^-05^ | 0.147 | 0.24±0.049 | 0.247±0.056 |
| cg07594831 | 8 | | *RNF19A* | 5'UTR;TSS200 | Open sea | 53.0 ± 4.7 | | 47.2 ± 4.8 | -5,7 | 6.71x10^-04^ | 0.212 | -0.362±0.08 | -0.324±0.092 |
| cg13675051 | 8 | | *RNF19A* | 5'UTR;TSS200 | Open sea | 38.2 ± 4.1 | | 32.6 ± 4.8 | -5,6 | 1.90x10^-04^ | 0.181 | -0.373±0.082 | -0.368±0.095 |
| cg13072943 | 6 | | *RPS6KA2* | Body | Open sea | 48.5 ± 3.3 | | 44.2 ± 4.3 | -4,2 | 4.05x10^-07^ | 0.082 | -0.308±0.057 | -0.366±0.064 |
| cg17501210 | 6 | | *RPS6KA2* | Body | Open sea | 70.8 ± 3.7 | | 66.9 ± 4.9 | -3,9 | 1.33x10^-03^ | 0.234 | -0.332±0.071 | -0.264±0.08 |
| cg11222173 | 17 | | *RPTOR* | Body | Open sea | 72.1 ± 3.4 | | 68.0 ± 3.9 | -4,1 | 1.26x10^-04^ | 0.179 | -0.333±0.073 | -0.339±0.084 |
| cg24419094 | 2 | | *RRM2* | Body | S Shelf | 58.3 ± 4.3 | | 53.1 ± 5.0 | -5,2 | 7.37x10^-05^ | 0.160 | -0.356±0.079 | -0.378±0.091 |
| cg08683249 | 6 | | *RSPH9* | TSS200 | N Shore | 69.7 ± 2.4 | | 66.7 ± 3.0 | -3 | 1.09x10^-03^ | 0.226 | -0.236±0.051 | -0.201±0.058 |
| cg06655349 | 19 | | *S1PR2* | 3'UTR | N Shelf | 44.3 ± 4.2 | | 38.9 ± 5.4 | -5,4 | 6.32x10^-04^ | 0.210 | -0.368±0.084 | -0.341±0.097 |
| cg13497089 | 4 | | *SCD5* | Body | N Shore | 64.7 ± 2.5 | | 61.5 ± 2.3 | -3,3 | 1.14x10^-04^ | 0.176 | -0.198±0.043 | -0.209±0.049 |
| cg11348106 | 17 | | *SEC14L1* | Body | N Shore | 75.6 ± 2.4 | | 78.4 ± 2.0 | 2,8 | 1.99x10^-05^ | 0.131 | 0.251±0.045 | 0.248±0.052 |
| cg27552857 | 19 | | *SEMA6B* | 3'UTR | N Shore | 50.7 ± 2.8 | | 47.4 ± 3.4 | -3,3 | 1.56x10^-04^ | 0.179 | -0.23±0.048 | -0.228±0.056 |
| cg10577241 | 15 | | *SGK269* | Body | Open sea | 66.5 ± 3.8 | | 71.2 ± 4.0 | 4,7 | 2.40x10^-04^ | 0.185 | 0.338±0.071 | 0.313±0.081 |
| cg26775087 | 3 | | *SH3BP5* | 1stExon;5'UTR | Open sea | 75.8 ± 2.4 | | 78.3 ± 2.1 | 2,5 | 1.06x10^-04^ | 0.175 | 0.244±0.052 | 0.25±0.06 |
| cg22291265 | 19 | | *SHANK1* | Body | Open sea | 7.5 ± 2.3 | | 5.8 ± 1.0 | -1,7 | 3.62x10^-05^ | 0.140 | -0.453±0.092 | -0.462±0.106 |
| cg18881723 | 1 | | *SLAMF1* | 5'UTR;1stExon | Open sea | 16.5 ± 2.9 | | 13.1 ± 2.2 | -3,5 | 1.84x10^-05^ | 0.131 | -0.418±0.08 | -0.421±0.092 |
| cg09001549 | 12 | | *SLC15A4* | Body | S Shore | 79.4 ± 4.4 | | 84.1 ± 3.4 | 4,7 | 1.12x10^-04^ | 0.175 | 0.479±0.11 | 0.509±0.127 |
| cg05200313 | 14 | | *SLC24A4* | 3'UTR | Open sea | 34.9 ± 3.7 | | 30.2 ± 5.0 | -4,6 | 2.32x10^-04^ | 0.185 | -0.36±0.082 | -0.362±0.095 |
| cg16104584 | 1 | | *SLC2A5* | TSS1500 | Open sea | 35.3 ± 3.6 | | 30.3 ± 5.1 | -5,1 | 1.88x10^-04^ | 0.181 | -0.362±0.077 | -0.348±0.089 |
| cg14176339 | 17 | | *SLC38A10* | Body | Open sea | 68.6 ± 3.5 | | 64.4 ± 2.9 | -4,2 | 3.57x10^-05^ | 0.140 | -0.31±0.057 | -0.293±0.065 |
| cg22897715 | 11 | | *SLC43A3* | Body | N Shelf | 12.6 ± 2.2 | | 10.1 ± 1.8 | -2,5 | 2.88x10^-05^ | 0.135 | -0.389±0.078 | -0.402±0.091 |
| cg23072823 | 3 | | *SLC6A6* | 5'UTR | S Shore | 13.6 ± 1.1 | | 11.9 ± 1.2 | -1,7 | 3.47x10^-04^ | 0.196 | -0.219±0.042 | -0.189±0.048 |
| cg02976539 | 17 | | *SLC9A3R1* | Body | Open sea | 67.6 ± 3.5 | | 70.7 ± 2.4 | 3,1 | 1.44x10^-04^ | 0.179 | 0.233±0.05 | 0.239±0.058 |
| cg04482712 | 17 | | *SLC9A3R1* | Body | Open sea | 71.5 ± 4.0 | | 74.5 ± 2.5 | 3 | 5.04x10^-04^ | 0.203 | 0.278±0.062 | 0.261±0.072 |
| cg02107844 | 15 | | *SLCO3A1* | Body | Open sea | 45.2 ± 2.3 | | 41.9 ± 3.1 | -3,3 | 8.03x10^-04^ | 0.218 | -0.208±0.045 | -0.186±0.051 |
| cg10876767 | 4 | | *SORCS2* | Body | Open sea | 41.1 ± 3.5 | | 36.5 ± 4.7 | -4,6 | 3.22x10^-05^ | 0.137 | -0.334±0.074 | -0.372±0.084 |
| cg11606261 | 12 | | *SP1* | 5'UTR;Body | S Shore | 32.3 ± 3.1 | | 27.5 ± 3.8 | -4,8 | 3.75x10^-05^ | 0.142 | -0.351±0.07 | -0.355±0.081 |
| cg07968760 | 12 | | *SPATS2* | 5'UTR | CpG Island | 55.1 ± 5.8 | | 51.3 ± 4.6 | -3,8 | 2.92x10^-03^ | 0.265 | -0.353±0.078 | -0.264±0.087 |
| cg03529189 | 12 | | *SRGAP1* | Body | Open sea | 55.6 ± 5.6 | | 49.0 ± 5.1 | -6,6 | 1.46x10^-04^ | 0.179 | -0.408±0.093 | -0.422±0.107 |
| cg25213452 | 1 | | *SRGAP2* | Body | Open sea | 58.4 ± 2.1 | | 55.4 ± 3.0 | -2,9 | 9.82x10^-05^ | 0.171 | -0.206±0.041 | -0.205±0.047 |
| cg12898019 | 17 | | *ST6GALNAC1* | 3'UTR | Open sea | 31.2 ± 4.2 | | 27.1 ± 3.6 | -4,1 | 2.74x10^-04^ | 0.187 | -0.308±0.069 | -0.304±0.08 |
| cg02961280 | 2 | | *STK16;TUBA4A* | Body;3'UTR | N Shelf | 19.5 ± 2.2 | | 16.6 ± 3.2 | -2,9 | 3.60x10^-04^ | 0.196 | -0.334±0.074 | -0.318±0.086 |
| cg18661379 | 10 | | *SUFU* | 3'UTR | Open sea | 21.0 ± 2.2 | | 17.5 ± 3.5 | -3,5 | 3.20x10^-04^ | 0.193 | -0.358±0.081 | -0.35±0.094 |
| cg11399254 | 1 | | *TAL1* | 5'UTR | N Shore | 50.8 ± 2.6 | | 47.2 ± 3.5 | -3,6 | 1.07x10^-05^ | 0.123 | -0.229±0.049 | -0.271±0.055 |
| cg11448683 | 1 | | *TCTEX1D4;BTBD19* | TSS1500;TSS200 | S Shore | 25.5 ± 3.1 | | 21.5 ± 3.6 | -4 | 3.28x10^-05^ | 0.137 | -0.344±0.067 | -0.342±0.077 |
| cg06589051 | 2 | | *TGFBRAP1* | TSS1500 | S Shore | 72.4 ± 2.0 | | 69.8 ± 3.2 | -2,6 | 1.44x10^-03^ | 0.238 | -0.229±0.05 | -0.191±0.056 |
| cg01649611 | 2 | | *THADA* | Body | Open sea | 19.1 ± 3.2 | | 16.0 ± 3.6 | -3,1 | 4.02x10^-04^ | 0.201 | -0.363±0.082 | -0.345±0.094 |
| cg23319460 | 11 | | *TIGD3* | Body | CpG Island | 61.9 ± 4.8 | | 57.5 ± 4.8 | -4,4 | 3.42x10^-04^ | 0.196 | -0.343±0.076 | -0.324±0.087 |
| cg26729380 | 6 | | *TNF* | 1stExon | Open sea | 17.4 ± 3.5 | | 14.4 ± 3.4 | -3 | 2.69x10^-05^ | 0.135 | -0.382±0.083 | -0.424±0.095 |
| cg08919597 | 6 | | *TNFAIP3* | Body | Open sea | 21.8 ± 3.1 | | 17.6 ± 3.1 | -4,2 | 5.29x10^-06^ | 0.109 | -0.465±0.078 | -0.441±0.09 |
| cg00524900 | 5 | | *TNFAIP8* | Body | Open sea | 32.1 ± 3.3 | | 27.6 ± 4.3 | -4,5 | 6.33x10^-04^ | 0.210 | -0.34±0.076 | -0.31±0.088 |
| cg08597832 | 8 | | *TOP1MT* | Body | N Shore | 82.1 ± 2.5 | | 78.9 ± 2.5 | -3,2 | 5.07x10^-05^ | 0.145 | -0.352±0.069 | -0.342±0.079 |
| cg00686823 | 3 | | *TPRA1* | TSS1500 | S Shore | 31.1 ± 4.5 | | 25.5 ± 4.6 | -5,6 | 8.84x10^-05^ | 0.167 | -0.461±0.097 | -0.458±0.112 |
| cg22644321 | 8 | | *TRIB1* | Body | S Shelf | 17.7 ± 2.5 | | 14.5 ± 2.6 | -3,2 | 5.74x10^-04^ | 0.206 | -0.358±0.079 | -0.324±0.091 |
| cg15022400 | 15 | | *TRIM69* | TSS1500 | Open sea | 22.0 ± 3.0 | | 18.7 ± 3.1 | -3,4 | 8.61x10^-04^ | 0.221 | -0.321±0.067 | -0.262±0.075 |
| cg00219303 | 22 | | *TRIOBP* | Body | S Shelf | 83.3 ± 2.7 | | 85.8 ± 1.6 | 2,5 | 1.32x10^-04^ | 0.179 | 0.29±0.065 | 0.302±0.075 |
| cg16509045 | 9 | | *TRPM6* | 5'UTR;1stExon | S Shore | 41.1 ± 4.3 | | 35.3 ± 5.0 | -5,9 | 7.64x10^-05^ | 0.163 | -0.401±0.085 | -0.407±0.098 |
| cg11166303 | 2 | | *TSSC1* | Body | N Shore | 67.8 ± 7.5 | | 61.9 ± 5.7 | -5,9 | 5.02x10^-05^ | 0.145 | -0.55±0.117 | -0.572±0.135 |
| cg01584932 | 2 | | *TTC31;CCDC142* | TSS1500;Body | N Shore | 40.9 ± 5.4 | | 35.2 ± 5.5 | -5,7 | 4.95x10^-06^ | 0.109 | -0.455±0.093 | -0.52±0.106 |
| cg04195000 | 22 | | *TTC38* | Body | S Shore | 80.2 ± 2.6 | | 82.8 ± 2.0 | 2,6 | 9.54x10^-04^ | 0.225 | 0.288±0.061 | 0.237±0.069 |
| cg02048220 | 14 | | *TTC7B* | Body | Open sea | 79.1 ± 2.2 | | 81.0 ± 2.2 | 1,9 | 5.71x10^-04^ | 0.206 | 0.241±0.053 | 0.225±0.061 |
| cg13709639 | 12 | | *TUBA1B* | TSS1500 | S Shore | 19.6 ± 2.8 | | 16.9 ± 3.1 | -2,7 | 2.22x10^-04^ | 0.185 | -0.32±0.07 | -0.314±0.081 |
| cg19513582 | 7 | | *UBE2H* | Body | Open sea | 49.9 ± 4.0 | | 44.6 ± 4.9 | -5,3 | 5.66x10^-05^ | 0.147 | -0.352±0.074 | -0.365±0.086 |
| cg02407068 | 4 | | *UBE2K* | Body | CpG Island | 90.5 ± 3.2 | | 93.5 ± 2.2 | 3,1 | 1.09x10^-04^ | 0.175 | 0.702±0.144 | 0.664±0.166 |
| cg03403155 | 8 | | *UBR5* | Body | N Shore | 28.0 ± 2.7 | | 24.6 ± 2.5 | -3,5 | 2.31x10^-05^ | 0.131 | -0.289±0.055 | -0.293±0.064 |
| cg12476487 | 6 | | *UTRN* | Body | Open sea | 73.9 ± 3.4 | | 77.0 ± 3.9 | 3,1 | 6.64x10^-04^ | 0.212 | 0.293±0.066 | 0.268±0.075 |
| cg10400707 | 1 | | *VANGL2* | TSS1500 | N Shore | 86.2 ± 2.5 | | 83.6 ± 2.5 | -2,6 | 1.69x10^-04^ | 0.180 | -0.354±0.079 | -0.358±0.091 |
| cg14710465 | 1 | | *VANGL2* | TSS1500 | N Shore | 95.5 ± 0.7 | | 94.8 ± 0.7 | -0,7 | 1.77x10^-04^ | 0.181 | -0.29±0.06 | -0.277±0.07 |
| cg03059896 | 1 | | *WDTC1* | TSS1500 | N Shore | 81.1 ± 3.0 | | 83.5 ± 1.6 | 2,4 | 1.81x10^-03^ | 0.247 | 0.272±0.059 | 0.214±0.066 |
| cg15876825 | 3 | | *VGLL4* | Body | Open sea | 94.8 ± 1.5 | | 96.5 ± 1.0 | 1,7 | 9.23x10^-06^ | 0.123 | 0.656±0.12 | 0.652±0.139 |
| cg04224247 | X | | *WWC3* | 5'UTR | CpG Island | 65.8 ± 10.0 | | 61.0 ± 8.5 | -4,8 | 1.44x10^-04^ | 0.179 | -0.487±0.095 | -0.429±0.109 |
| cg25270201 | X | | *WWC3* | 5'UTR | CpG Island | 28.5 ± 14.7 | | 21.6 ± 14.0 | -6,9 | 3.80x10^-04^ | 0.197 | -0.392±0.079 | -0.33±0.09 |
| cg21775279 | 1 | | *XKR8;SMPDL3B* | TSS1500;3'UTR | N Shore | 85.7 ± 1.8 | | 83.2 ± 2.2 | -2,5 | 1.42x10^-04^ | 0.179 | -0.296±0.06 | -0.28±0.069 |
| cg26730763 | 16 | | *XPO6* | Body | Open sea | 13.2 ± 2.1 | | 10.9 ± 2.3 | -2,3 | 1.67x10^-04^ | 0.180 | -0.384±0.084 | -0.378±0.097 |
| cg00805874 | 6 | | *ZBTB12* | Body | CpG Island | 71.4 ± 5.7 | | 78.1 ± 5.3 | 6,6 | 1.54x10^-04^ | 0.179 | 0.557±0.122 | 0.55±0.141 |
| cg08975528 | 6 | | *ZBTB12* | 3'UTR | CpG Island | 62.5 ± 22.9 | | 76.0 ± 6.1 | 13,5 | 1.02x10^-03^ | 0.226 | 1.379±0.313 | 1.182±0.358 |
| cg09788778 | 6 | | *ZBTB12* | Body | CpG Island | 71.5 ± 5.7 | | 77.3 ± 4.9 | 5,8 | 5.27x10^-04^ | 0.203 | 0.483±0.111 | 0.453±0.128 |
| cg25470384 | 6 | | *ZBTB12* | Body | CpG Island | 88.0 ± 3.7 | | 91.7 ± 2.4 | 3,7 | 3.87x10^-05^ | 0.145 | 0.648±0.128 | 0.635±0.148 |
| cg13307142 | X | | *ZDHHC15* | 5'UTR;1stExon | CpG Island | 23.0 ± 21.1 | | 17.6 ± 19.8 | -5,4 | 9.57x10^-05^ | 0.170 | -0.232±0.051 | -0.246±0.058 |
| cg00602811 | 2 | | *ZEB2* | TSS1500 | N Shelf | 43.4 ± 5.6 | | 38.5 ± 5.8 | -4,9 | 2.67x10^-04^ | 0.187 | -0.346±0.074 | -0.323±0.085 |
| cg03743205 | 16 | | *ZFPM1* | Body | CpG Island | 17.7 ± 3.7 | | 14.1 ± 3.3 | -3,6 | 1.87x10^-04^ | 0.181 | -0.466±0.103 | -0.461±0.119 |
| cg23084416 | 10 | | *ZMIZ1* | 5'UTR | Open sea | 73.2 ± 3.2 | | 70.6 ± 3.3 | -2,5 | 2.37x10^-03^ | 0.256 | -0.24±0.051 | -0.18±0.056 |
| cg00852033 | 16 | | *ZNF598* | Body | N Shore | 8.2 ± 0.8 | | 7.3 ± 0.8 | -0,9 | 6.53x10^-06^ | 0.109 | -0.226±0.042 | -0.246±0.048 |
| cg07643930 | 16 | | *ZNF598* | TSS1500 | S Shore | 16.6 ± 2.4 | | 14.6 ± 2.2 | -1,9 | 2.69x10^-04^ | 0.187 | -0.263±0.057 | -0.257±0.066 |
| cg00454592 | 2 | |  | Intergenic | Open sea | 57.0 ± 4.3 | | 50.7 ± 5.3 | -6,3 | 6.03x10^-06^ | 0.109 | -0.415±0.081 | -0.450±0.093 |
| cg00673344 | 3 | |  | Intergenic | S Shore | 30.7 ± 3.1 | | 26.4 ± 3.5 | -4,3 | 7.23x10^-05^ | 0.160 | -0.332±0.066 | -0.322±0.076 |
| cg00905524 | 7 | |  | Intergenic | N Shore | 17.2 ± 3.4 | | 13.9 ± 2.6 | -3,3 | 5.53x10^-05^ | 0.147 | -0.425±0.09 | -0.437±0.103 |
| cg01764252 | 5 | |  | Intergenic | Open sea | 14.8 ± 2.1 | | 13.1 ± 1.5 | -1,7 | 5.08x10^-04^ | 0.203 | -0.28±0.057 | -0.236±0.064 |
| cg01879591 | 2 | |  | Intergenic | S Shore | 42.0 ± 5.5 | | 35.6 ± 5.7 | -6,4 | 1.72x10^-04^ | 0.181 | -0.439±0.095 | -0.428±0.11 |
| cg02030958 | 13 | |  | Intergenic | Open sea | 71.7 ± 4.6 | | 77.7 ± 5.7 | 6 | 7.98x10^-05^ | 0.167 | 0.56±0.121 | 0.574±0.14 |
| cg02060682 | 7 | |  | Intergenic | N Shore | 55.7 ± 3.5 | | 50.3 ± 4.2 | -5,4 | 5.03x10^-06^ | 0.109 | -0.301±0.065 | -0.362±0.073 |
| cg03332892 | X | |  | Intergenic | N Shelf | 75.3 ± 11.2 | | 74.6 ± 11.7 | -0,7 | 2.49x10^-05^ | 0.135 | -0.295±0.058 | -0.306±0.067 |
| cg03486991 | 2 | |  | Intergenic | N Shore | 46.1 ± 2.8 | | 41.7 ± 3.5 | -4,3 | 2.11x10^-04^ | 0.183 | -0.272±0.054 | -0.246±0.062 |
| cg03889263 | 3 | |  | Intergenic | Open sea | 20.0 ± 3.9 | | 16.3 ± 3.0 | -3,8 | 7.19x10^-05^ | 0.160 | -0.429±0.097 | -0.461±0.111 |
| cg05428701 | 11 | |  | Intergenic | S Shore | 17.6 ± 1.9 | | 15.3 ± 1.8 | -2,3 | 2.19x10^-05^ | 0.131 | -0.251±0.053 | -0.282±0.061 |
| cg05546763 | 14 | |  | Intergenic | CpG Island | 82.0 ± 2.0 | | 80.2 ± 2.1 | -1,8 | 2.86x10^-05^ | 0.135 | -0.245±0.044 | -0.238±0.051 |
| cg06126421 | 6 | |  | Intergenic | Open sea | 69.2 ± 6.2 | | 61.1 ± 9.5 | -8,1 | 4.26x10^-04^ | 0.202 | -0.622±0.124 | -0.5±0.139 |
| cg06393679 | 7 | |  | Intergenic | Open sea | 80.8 ± 1.8 | | 82.4 ± 1.2 | 1,6 | 9.40x10^-04^ | 0.225 | 0.18±0.038 | 0.159±0.044 |
| cg06407843 | 6 | |  | Intergenic | S Shelf | 41.8 ± 4.5 | | 35.7 ± 5.9 | -6,1 | 2.57x10^-04^ | 0.187 | -0.4±0.092 | -0.401±0.106 |
| cg06532546 | 1 | |  | Intergenic | CpG Island | 74.7 ± 6.6 | | 80.8 ± 5.2 | 6,1 | 7.84x10^-04^ | 0.217 | 0.631±0.145 | 0.568±0.167 |
| cg07948143 | 14 | |  | Intergenic | Open sea | 75.5 ± 3.2 | | 72.2 ± 2.9 | -3,4 | 6.81x10^-05^ | 0.160 | -0.319±0.065 | -0.319±0.075 |
| cg08155249 | 5 | |  | Intergenic | N Shore | 54.4 ± 2.9 | | 50.7 ± 3.6 | -3,7 | 1.56x10^-04^ | 0.179 | -0.238±0.053 | -0.248±0.061 |
| cg08763886 | 11 | |  | Intergenic | Open sea | 87.8 ± 1.4 | | 86.5 ± 1.0 | -1,3 | 4.87x10^-05^ | 0.145 | -0.218±0.043 | -0.223±0.049 |
| cg09298313 | 14 | |  | Intergenic | Open sea | 35.9 ± 2.8 | | 32.3 ± 2.9 | -3,6 | 4.19x10^-07^ | 0.082 | -0.273±0.046 | -0.305±0.052 |
| cg09340403 | 6 | |  | Intergenic | Open sea | 55.2 ± 2.6 | | 51.6 ± 3.3 | -3,5 | 4.64x10^-05^ | 0.145 | -0.233±0.05 | -0.254±0.057 |
| cg09396865 | 2 | |  | Intergenic | Open sea | 50.0 ± 3.7 | | 44.2 ± 4.7 | -5,8 | 4.27x10^-06^ | 0.109 | -0.344±0.072 | -0.404±0.081 |
| cg09577317 | 8 | |  | Intergenic | N Shore | 83.9 ± 1.6 | | 81.8 ± 1.9 | -2 | 4.43x10^-05^ | 0.145 | -0.237±0.049 | -0.254±0.057 |
| cg09920072 | 2 | |  | Intergenic | S Shore | 83.6 ± 1.8 | | 85.3 ± 1.3 | 1,6 | 1.30x10^-04^ | 0.179 | 0.207±0.045 | 0.216±0.051 |
| cg11074232 | 2 | |  | Intergenic | S Shore | 25.2 ± 2.1 | | 22.9 ± 2.2 | -2,3 | 5.26x10^-05^ | 0.145 | -0.226±0.047 | -0.239±0.054 |
| cg11481582 | 10 | |  | Intergenic | S Shore | 26.0 ± 3.4 | | 22.0 ± 2.9 | -3,9 | 3.61x10^-04^ | 0.196 | -0.338±0.074 | -0.317±0.085 |
| cg11960708 | 6 | |  | Intergenic | S Shore | 89.2 ± 1.2 | | 90.2 ± 1.2 | 1 | 4.05x10^-03^ | 0.286 | 0.212±0.046 | 0.156±0.051 |
| cg12109883 | 15 | |  | Intergenic | Open sea | 65.8 ± 2.3 | | 63.6 ± 2.7 | -2,1 | 1.35x10^-04^ | 0.179 | -0.188±0.04 | -0.197±0.046 |
| cg13069322 | 7 | |  | Intergenic | Open sea | 34.4 ± 4.2 | | 28.7 ± 4.7 | -5,7 | 1.09x10^-04^ | 0.175 | -0.4±0.086 | -0.403±0.1 |
| cg13488811 | 11 | |  | Intergenic | Open sea | 48.5 ± 5.8 | | 42.8 ± 4.6 | -5,7 | 3.49x10^-05^ | 0.140 | -0.416±0.087 | -0.436±0.1 |
| cg13645242 | 7 | |  | Intergenic | N Shore | 85.8 ± 1.7 | | 83.9 ± 1.4 | -1,9 | 1.09x10^-05^ | 0.123 | -0.268±0.048 | -0.27±0.055 |
| cg13873263 | 17 | |  | Intergenic | N Shelf | 46.9 ± 2.8 | | 43.8 ± 3.7 | -3,1 | 1.52x10^-05^ | 0.126 | -0.236±0.05 | -0.272±0.057 |
| cg13984040 | 12 | |  | Intergenic | Open sea | 32.8 ± 4.4 | | 28.3 ± 5.5 | -4,5 | 6.15x10^-04^ | 0.210 | -0.358±0.079 | -0.319±0.09 |
| cg14003416 | 2 | |  | Intergenic | Open sea | 45.3 ± 4.0 | | 40.0 ± 4.3 | -5,4 | 2.73x10^-06^ | 0.102 | -0.359±0.071 | -0.412±0.081 |
| cg14011327 | 4 | |  | Intergenic | Open sea | 13.7 ± 2.2 | | 11.1 ± 1.6 | -2,6 | 8.96x10^-06^ | 0.123 | -0.386±0.071 | -0.392±0.082 |
| cg14205216 | 6 | |  | Intergenic | Open sea | 69.4 ± 3.8 | | 66.0 ± 3.2 | -3,4 | 8.29x10^-05^ | 0.167 | -0.298±0.066 | -0.316±0.076 |
| cg14577707 | 4 | |  | Intergenic | Open sea | 60.6 ± 6.1 | | 55.5 ± 7.0 | -5 | 1.44x10^-04^ | 0.179 | -0.439±0.088 | -0.4±0.101 |
| cg14622879 | 6 | |  | Intergenic | Open sea | 20.9 ± 3.0 | | 16.8 ± 3.3 | -4,1 | 2.54x10^-06^ | 0.102 | -0.443±0.077 | -0.455±0.089 |
| cg14753356 | 6 | |  | Intergenic | Open sea | 42.4 ± 3.9 | | 37.4 ± 5.5 | -5 | 5.10x10^-04^ | 0.203 | -0.356±0.076 | -0.314±0.087 |
| cg15034393 | 3 | |  | Intergenic | Open sea | 36.6 ± 3.8 | | 33.3 ± 4.5 | -3,3 | 8.59x10^-05^ | 0.167 | -0.295±0.06 | -0.291±0.069 |
| cg15342087 | 6 | |  | Intergenic | Open sea | 81.1 ± 2.1 | | 78.4 ± 3.5 | -2,7 | 3.75x10^-03^ | 0.282 | -0.291±0.061 | -0.197±0.065 |
| cg15989436 | 5 | |  | Intergenic | Open sea | 37.8 ± 3.4 | | 32.9 ± 3.7 | -4,9 | 8.66x10^-05^ | 0.167 | -0.334±0.063 | -0.303±0.072 |
| cg16149628 | 11 | |  | Intergenic | Open sea | 59.6 ± 4.9 | | 53.7 ± 6.1 | -5,9 | 1.91x10^-04^ | 0.181 | -0.409±0.091 | -0.409±0.106 |
| cg16579650 | 10 | |  | Intergenic | Open sea | 77.6 ± 2.0 | | 75.0 ± 1.5 | -2,6 | 1.09x10^-04^ | 0.175 | -0.202±0.041 | -0.205±0.048 |
| cg16711650 | 1 | |  | Intergenic | Open sea | 19.8 ± 2.7 | | 16.7 ± 2.9 | -3 | 6.74x10^-04^ | 0.212 | -0.33±0.075 | -0.303±0.086 |
| cg17380244 | 2 | |  | Intergenic | Open sea | 79.7 ± 2.8 | | 78.1 ± 2.4 | -1,6 | 2.83x10^-04^ | 0.190 | -0.25±0.055 | -0.245±0.063 |
| cg17622952 | 11 | |  | Intergenic | Open sea | 74.0 ± 3.7 | | 69.6 ± 3.9 | -4,5 | 3.96x10^-04^ | 0.200 | -0.361±0.079 | -0.334±0.091 |
| cg17759224 | 1 | |  | Intergenic | Open sea | 27.5 ± 2.6 | | 23.9 ± 3.7 | -3,7 | 1.61x10^-04^ | 0.180 | -0.314±0.067 | -0.307±0.077 |
| cg18173184 | 12 | |  | Intergenic | S Shore | 33.2 ± 4.1 | | 28.6 ± 4.3 | -4,6 | 2.24x10^-04^ | 0.185 | -0.372±0.081 | -0.358±0.093 |
| cg18764008 | 5 | |  | Intergenic | Open sea | 65.9 ± 3.2 | | 63.4 ± 2.7 | -2,5 | 2.35x10^-04^ | 0.185 | -0.242±0.048 | -0.22±0.055 |
| cg18887769 | 14 | |  | Intergenic | Open sea | 69.2 ± 3.2 | | 72.9 ± 2.8 | 3,7 | 3.94x10^-05^ | 0.145 | 0.281±0.056 | 0.285±0.064 |
| cg18925366 | 14 | |  | Intergenic | Open sea | 91.2 ± 1.2 | | 90.1 ± 1.3 | -1,1 | 4.88x10^-05^ | 0.145 | -0.259±0.055 | -0.277±0.063 |
| cg19017142 | 2 | |  | Intergenic | Open sea | 45.3 ± 3.4 | | 42.0 ± 4.6 | -3,3 | 3.85x10^-04^ | 0.198 | -0.292±0.061 | -0.261±0.07 |
| cg19270739 | 1 | |  | Intergenic | N Shore | 43.2 ± 4.0 | | 40.3 ± 3.7 | -2,8 | 2.08x10^-06^ | 0.102 | -0.218±0.048 | -0.28±0.052 |
| cg19770281 | 3 | |  | Intergenic | Open sea | 31.1 ± 3.7 | | 26.4 ± 4.5 | -4,7 | 1.45x10^-04^ | 0.179 | -0.394±0.083 | -0.381±0.096 |
| cg19925518 | 10 | |  | Intergenic | Open sea | 60.5 ± 4.9 | | 55.0 ± 4.8 | -5,5 | 7.93x10^-06^ | 0.120 | -0.391±0.082 | -0.448±0.094 |
| cg20706315 | 17 | |  | Intergenic | Open sea | 77.3 ± 1.9 | | 74.3 ± 2.5 | -2,9 | 4.96x10^-06^ | 0.109 | -0.249±0.048 | -0.282±0.055 |
| cg20954870 | 5 | |  | Intergenic | Open sea | 46.8 ± 4.9 | | 40.9 ± 5.5 | -6 | 6.79x10^-05^ | 0.160 | -0.388±0.088 | -0.424±0.102 |
| cg21153342 | 3 | |  | Intergenic | Open sea | 79.0 ± 2.9 | | 75.2 ± 3.2 | -3,8 | 3.97x10^-04^ | 0.200 | -0.324±0.072 | -0.305±0.083 |
| cg21526750 | 14 | |  | Intergenic | CpG Island | 17.7 ± 4.0 | | 14.6 ± 4.0 | -3,2 | 3.89x10^-05^ | 0.145 | -0.513±0.118 | -0.581±0.135 |
| cg23159704 | 20 | |  | Intergenic | CpG Island | 63.9 ± 4.7 | | 59.7 ± 4.5 | -4,2 | 6.30x10^-04^ | 0.210 | -0.331±0.075 | -0.305±0.086 |
| cg23539261 | 12 | |  | Intergenic | Open sea | 59.8 ± 2.7 | | 56.7 ± 2.6 | -3,1 | 6.58x10^-05^ | 0.159 | -0.223±0.044 | -0.225±0.051 |
| cg24025721 | 7 | |  | Intergenic | Open sea | 50.0 ± 3.7 | | 45.3 ± 4.9 | -4,7 | 5.81x10^-05^ | 0.149 | -0.314±0.071 | -0.346±0.081 |
| cg24058013 | 18 | |  | Intergenic | CpG Island | 89.8 ± 1.0 | | 88.1 ± 1.5 | -1,6 | 5.11x10^-07^ | 0.082 | -0.232±0.05 | -0.31±0.054 |
| cg24254488 | 7 | |  | Intergenic | S Shore | 41.6 ± 4.4 | | 36.1 ± 5.1 | -5,5 | 8.53x10^-06^ | 0.121 | -0.402±0.08 | -0.44±0.092 |
| cg24315421 | 1 | |  | Intergenic | N Shore | 21.4 ± 3.3 | | 17.4 ± 3.5 | -4 | 1.18x10^-04^ | 0.176 | -0.42±0.093 | -0.432±0.108 |
| cg24400656 | 6 | |  | Intergenic | Open sea | 40.0 ± 4.3 | | 35.0 ± 4.1 | -5 | 3.60x10^-04^ | 0.196 | -0.353±0.074 | -0.317±0.085 |
| cg24430034 | 13 | |  | Intergenic | Open sea | 86.7 ± 3.2 | | 90.6 ± 3.1 | 4 | 1.25x10^-05^ | 0.123 | 0.701±0.132 | 0.7±0.152 |
| cg24834394 | 12 | |  | Intergenic | Open sea | 27.1 ± 3.1 | | 23.6 ± 3.3 | -3,5 | 3.41x10^-05^ | 0.140 | -0.317±0.068 | -0.346±0.078 |
| cg25278941 | 6 | |  | Intergenic | Open sea | 33.2 ± 4.0 | | 28.7 ± 5.3 | -4,5 | 1.99x10^-05^ | 0.131 | -0.384±0.076 | -0.401±0.088 |
| cg25494075 | 11 | |  | Intergenic | Open sea | 83.0 ± 1.8 | | 85.2 ± 1.5 | 2,2 | 1.76x10^-05^ | 0.131 | 0.254±0.052 | 0.281±0.06 |
| cg25771113 | 3 | |  | Intergenic | Open sea | 31.5 ± 3.8 | | 27.3 ± 4.6 | -4,2 | 1.32x10^-04^ | 0.179 | -0.34±0.077 | -0.356±0.089 |
| cg25921758 | 4 | |  | Intergenic | Open sea | 74.0 ± 3.3 | | 77.1 ± 3.0 | 3,1 | 2.29x10^-05^ | 0.131 | 0.308±0.064 | 0.335±0.074 |
| cg25975690 | 7 | |  | Intergenic | CpG Island | 33.5 ± 3.3 | | 29.4 ± 3.3 | -4 | 8.76x10^-05^ | 0.167 | -0.311±0.063 | -0.302±0.072 |
| cg26101890 | 2 | |  | Intergenic | Open sea | 90.2 ± 2.4 | | 92.7 ± 1.5 | 2,5 | 1.79x10^-04^ | 0.181 | 0.487±0.106 | 0.473±0.122 |
| cg26140475 | 8 | |  | Intergenic | Open sea | 23.2 ± 2.8 | | 20.1 ± 3.7 | -3,1 | 1.21x10^-04^ | 0.177 | -0.332±0.072 | -0.338±0.084 |
| cg26427498 | 7 | |  | Intergenic | Open sea | 20.1 ± 3.8 | | 15.9 ± 3.4 | -4,2 | 1.14x10^-05^ | 0.123 | -0.472±0.088 | -0.473±0.101 |
| cg26856257 | 1 | |  | Intergenic | S Shelf | 20.7 ± 2.9 | | 17.4 ± 2.9 | -3,3 | 3.15x10^-04^ | 0.193 | -0.325±0.069 | -0.298±0.079 |
| cg27031754 | 5 | |  | Intergenic | Open sea | 67.9 ± 2.5 | | 70.7 ± 2.5 | 2,8 | 7.29x10^-05^ | 0.160 | 0.216±0.046 | 0.229±0.053 |
| cg27040968 | 14 | |  | Intergenic | Open sea | 77.2 ± 2.1 | | 79.4 ± 2.1 | 2,1 | 3.21x10^-04^ | 0.193 | 0.224±0.049 | 0.219±0.057 |
| cg27208467 | X | |  | Intergenic | S Shore | 79.8 ± 13.0 | | 79.8 ± 14.1 | 0,1 | 1.19x10^-04^ | 0.176 | -0.361±0.074 | -0.344±0.085 |
